# Cellular and molecular properties of neural progenitors in the developing mammalian hypothalamus

**DOI:** 10.1038/s41467-020-17890-2

**Published:** 2020-08-13

**Authors:** Xin Zhou, Suijuan Zhong, Honghai Peng, Jing Liu, Wenyu Ding, Le Sun, Qiang Ma, Zeyuan Liu, Ruiguo Chen, Qian Wu, Xiaoqun Wang

**Affiliations:** 1 State Key Laboratory of Brain and Cognitive Science, CAS Center for Excellence in Brain Science and Intelligence Technology, Institute of Brain-Intelligence Technology (Shanghai), Bioland Laboratory (Guangzhou), Institute of Biophysics, Chinese Academy of Sciences, Beijing, 100101 China; 2 State Key Laboratory of Cognitive Neuroscience and Learning, Beijing Normal University, Beijing, 100875 China; 3 IDG/McGovern Institute for Brain Research, Beijing Normal University, Beijing, 100875 China; 4 Department of Neurosurgery, Jinan Central Hospital Affiliated to Shandong University, Shandong, 250013 China; 5 University of Chinese Academy of Sciences, Beijing, 100049 China; 6 Institute for Stem Cell and Regeneration, Chinese Academy of Sciences, Beijing, 100101 China; 7 Advanced Innovation Center for Human Brain Protection, Beijing Institute for Brain Disorders, Capital Medical University, Beijing, 100069 China

**Keywords:** Developmental neurogenesis, Neural progenitors

## Abstract

The neuroendocrine hypothalamus is the central regulator of vital physiological homeostasis and behavior. However, the cellular and molecular properties of hypothalamic neural progenitors remain unexplored. Here, hypothalamic radial glial (hRG) and hypothalamic mantle zone radial glial (hmRG) cells are found to be neural progenitors in the developing mammalian hypothalamus. The hmRG cells originate from hRG cells and produce neurons. During the early development of hypothalamus, neurogenesis occurs in radial columns and is initiated from hRG cells. The radial glial fibers are oriented toward the locations of hypothalamic subregions which act as a scaffold for neuronal migration. Furthermore, we use single-cell RNA sequencing to reveal progenitor subtypes in human developing hypothalamus and characterize specific progenitor genes, such as *TTYH1*, *HMGA2*, and *FAM107A*. We also demonstrate that HMGA2 is involved in E2F1 pathway, regulating the proliferation of progenitor cells by targeting on the downstream MYBL2. Different neuronal subtypes start to differentiate and express specific genes of hypothalamic nucleus at gestational week 10. Finally, we reveal the developmental conservation of nuclear structures and marker genes in mouse and human hypothalamus. Our identification of cellular and molecular properties of neural progenitors provides a basic understanding of neurogenesis and regional formation of the non-laminated hypothalamus.

## Abstract (web summary)

The hypothalamus performs a wide range of vital physiological functions, including growth and reproductive behaviors, and circadian rhythms. The authors identify and characterize hypothalamic radial glial and hypothalamic mantle zone radial glial cells as the neural progenitors in the hypothalamus.

## Introduction

The hypothalamus, which is located ventral to the thalamus, is an evolutionarily ancient portion of the brain, and some neuronal subtypes in this structure are functionally conserved across different vertebrate species^1–6^. The hypothalamus is essential for homeostasis and energy metabolism^2,4^, and defects in its development lead to diseases or disorders in adulthood, such as energy imbalance, obesity, anxiety, etc.^7–11^.

In contrast to the cortex that is organized in a columnar structure, hypothalamic neurons form multiple functional nuclei arrayed in a three-dimensional structure to regulate behavior^2–4^. Each hypothalamic nucleus is distinguished by its location in the rostral–caudal axis and plays specific or overlapping roles in regulating homeostasis in vertebrates^2,8^. For example, neurons responsive to nutrient-related signals and hormones such as leptin, glucose, and insulin in the ventromedial hypothalamus (VMH) have been reported to regulate feeding behavior^12–14^. In addition, the arcuate nucleus (ARC) contains two subpopulation of neurons, pro-opiomelanocortin (POMC) neurons and agouti-related protein neurons, which control energy balance^15,16^. The diverse functions are supported by molecularly specialized subtypes of neurons located within these distinct nuclei. Previous genetic analysis studies have shown that individual hypothalamic neuron subpopulation in different nuclei selectively controlled specific fundamental behaviors^16–19^. Moreover, recent work has made progress to profile the transcriptome of distinct cell types in certain hypothalamic regions by single-cell RNA sequencing (scRNA-seq)^20–24^. Hence, an understanding of the mechanisms by which hypothalamic neurons are generated and position themselves into nuclei during development would shed light on the functional diversity and complexity of different hypothalamic nuclei. However, little is known about cellular properties and molecular identities of hypothalamic neural progenitor cells.

Here we define the neural progenitor cells in the developing mammalian hypothalamus. Other than traditional hypothalamic radial glial (hRG) cells, which are predominantly located in the ventricular zone (VZ) in proximity to the third ventricle, we found RG-like cells with only a basal process in mantle zone (MZ), which are named as hypothalamic mantle zone radial glial (hmRG) cells. Time-lapse imaging reveals that hRG and hmRG cells undergo interkinetic nuclear migration (INM) and mitotic somal translocation (MST), respectively, during cell divisions. The hmRG cells are generated from hRG cells and functioned as neural progenitors to produce neurons. Progenitors in the MZ divide symmetrically to generate daughter cells and are defined as MZ progenitor cells. The RG fibers oriented toward the presumptive hypothalamic nuclei act as scaffolds for migrating neurons. Using scRNA-seq, we define different clusters of progenitors and neurons with distinct transcriptional signatures. We identify specific marker genes expressed in different subtypes of progenitor cells in the human hypothalamus. Particularly, progenitor subtypes characterized as hRG are predicted to generate diverse neuronal progenies that contribute to multiple nuclei formation. Despite of shared features by hmRG in hypothalamus and outer RG (oRG) in the cortex, we illustrate different gene expression pattern between them. The co-expression module analysis reveals that E2F1 regulates the proliferation of hypothalamic progenitors (HPCs) by acting on the downstream MYBL2. In addition, our data also reveal that the transcription factors involved in the neuronal differentiation and formation of hypothalamic nuclei are highly conserved in mammals. Thus this study reveals the cellular and molecular features of different types of hypothalamic neural progenitor cells, elaborating the mechanism of hypothalamic neurogenesis.

## Results

### Progenitors in the developing mammalian hypothalamus

**Fig. 1:**
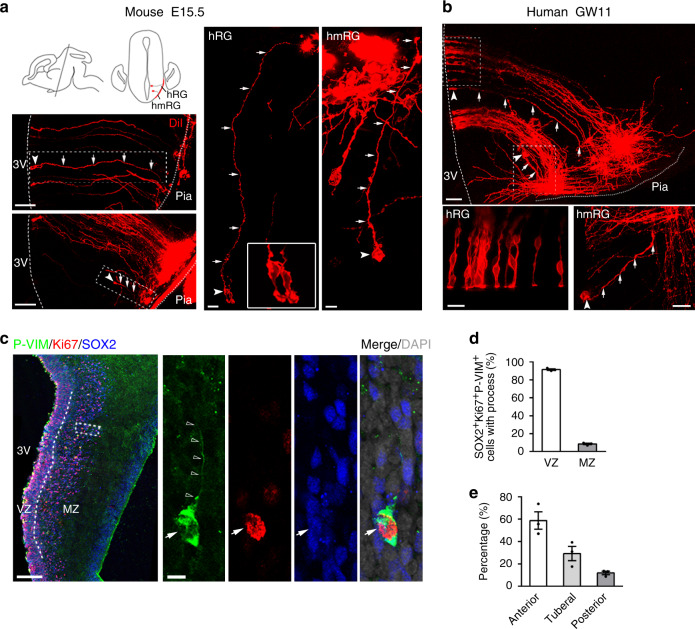
Progenitor composition in the developing mammalian hypothalamus. **a**, **b** The mouse and human hypothalamic VZ and MZ are populated with radial glia and monopolar radial glia-like cells. The morphology of hypothalamic progenitors was labeled with DiI crystals on the pial surface. The insert shows dye diffusion along radial fibers (arrow) from the hypothalamic pia terminating at distinct cell bodies (arrowhead) in the MZ or along the radial fibers traversing to the radial glia cells in the VZ at E15.5 in mice (**a**, *n* = 6 independent experiments) and GW11 in humans (**b**, *n* = 3 independent experiments). Dashed lines indicate the surface of the third ventricle. Dotted lines indicate the pial surface. 3V, the third ventricle. Scale bars, 100 µm (left, top and bottom), 20 µm (middle), 10 µm (right) in **a**, 50 µm (top) and 20 µm (bottom) in **b**. **c** P-VIM^+^ (green) hmRG cells in the GW10 human hypothalamus were stained for SOX2 (blue) and Ki67 (red). High-magnification images of the representative outlined cell are shown in the right panels. Arrows indicate hmRG cells that co-expressed Ki67 (red) and SOX2 (blue). Open arrowheads indicate the basal processes. Scale bars, 200 μm (left) and 10 μm (right). **d** Quantification of the percentage of RG cells identified by SOX2^+^Ki67^+^P-VIM^+^ immunostaining in the VZ and MZ (*n* = 3 independent experiments). **e** Quantification of the percentage of hmRG cells (SOX2^+^Ki67^+^P-VIM^+^) in the MZ from the anterior to posterior hypothalamus (*n* = 3 independent experiments). Data are presented as mean values ± SEM. Source data are supplied as a Source Data file.

In the developing mammalian neocortex, neural progenitor types are well established^25–30^. However, the molecular and cellular mechanisms underlying the neurogenesis and organization of hypothalamic nuclei remain largely unknown. Hence, we applied DiI crystals to the pial surface of the fixed mouse (embryonic day 13.5 (E13.5) and E15.5) and human (gestational week 11 (GW11)) hypothalamus to label the shape of cells that attached to the pia (Fig. 1a, b, and Supplementary Fig. 1a, b). DiI-labeled cells in the VZ showed a typical neocortical RG-like morphology, with a short apical process extending to the VZ surface with an endfoot and a long fine basal process protruding to the pial surface in the developing mouse and human hypothalamus. We refer to these cells as hRG cells (Fig. 1a, b and Supplementary Fig. 1a, b). Ring-like structures, as viewed by staining for junctional proteins such as zona occludens 1 (ZO-1), β-catenin, and N-cadherin, existed in the endfeet of hRG cells to maintain apical attachments to the luminal surface of the VZ (Supplementary Fig. 1c).

Other than hRG, we also observed that RG-like cells, localized in mantle zone, only produced basal processes that contacted the pia but no apical processes in the mouse and human hypothalamus. Hence, we refer to these cells as hmRG cells (Fig. 1a, b and Supplementary Fig. 1a). To identify whether these cells are progenitors, we electroporated enhanced green fluorescent protein (EGFP)-expressing plasmids into the third ventricle of mice at E13.5 and observed SOX2^+^ staining in EGFP^+^ hRG and hmRG cells in the hypothalamic tissue at E15.5 (Supplementary Fig. 1d). The hRG cells were located in the VZ, while hmRG cells were dispersed throughout the hypothalamic MZ (Supplementary Fig. 1d). We next stained the sections for phospho-vimentin (P-VIM) and Ki67 as proliferation markers and SOX2 as a progenitor marker to further characterize hmRG cells. SOX2^+^Ki67^+^P-VIM^+^ hmRG cells were sparsely distributed in the hypothalamic MZ, some of which were located close to the VZ, which was designated as the medial MZ in the developing human hypothalamus at GW10 and E13.5 mouse hypothalamus (Fig. 1c and Supplementary Fig. 1e). Approximately 91% of triple-positive progenitor cells were located in the VZ of the mouse hypothalamus, suggesting that hRG cells were the major progenitors at E13.5 (Fig. 1d). The hmRG cells exhibited a distribution with an anterior to posterior spatial gradient, and 58.71%, 29.32%, and 11.96% of hmRG cells were located in the anterior, tuberal, and posterior mouse hypothalamus, respectively (Fig. 1e and Supplementary Fig. 1e). Taken together, the results indicate that the developing mammalian hypothalamus contain traditional RG cells in the VZ and newly found RG-like cells in MZ that are progressing through the cell cycle.

### RG cells are neuronal progenitors

**Fig. 2:**
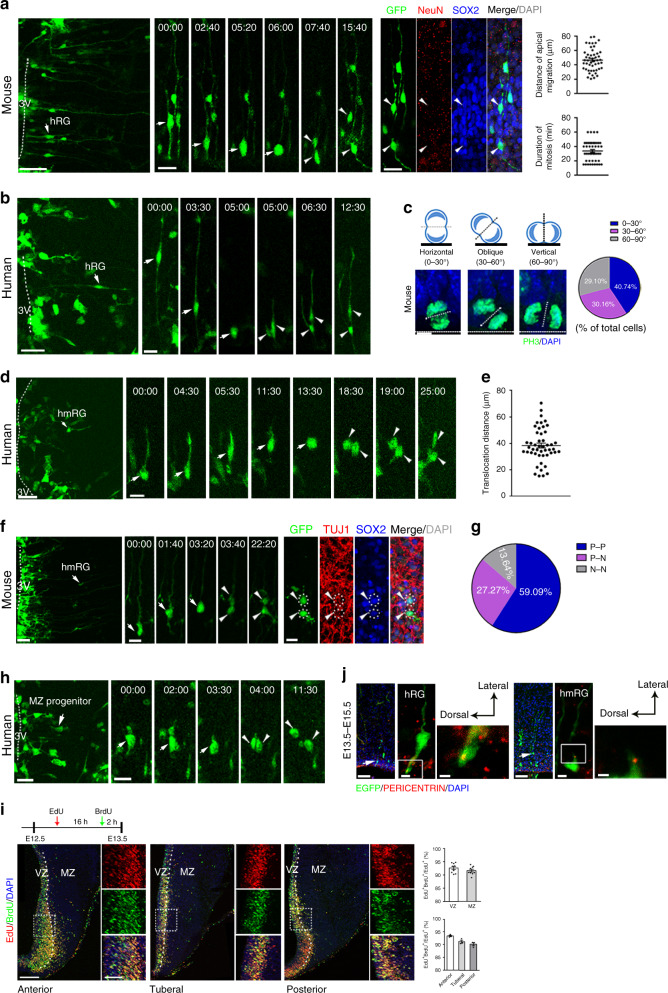
Hypothalamic progenitors undergo classical division behavior. **a** Adeno-GFP-labeled RGs (arrow) undergo INM and divide at the ventricular surface. An hmRG daughter cell (arrowhead) was SOX2^+^. Measurements of the distance of apical migration and mitotic duration (*n* = 49 cells). Scale bars, 50 μm (left) and 20 μm (middle and right). **b** Time-lapse imaging of human hypothalamic radial glia (*n* = 3 independent experiments). Dashed lines indicate the third ventricle. Time stamp, h:min. Scale bars, 50 μm (left) and 20 μm (right). **c** Measurement of the percentage of cells displaying a particular cleavage plane orientation at E13.5. **d** Time-lapse imaging of hmRGs labeled with Adeno-GFP in human hypothalamic slices. Time stamp, h:min. Scale bars, 50 μm (left) and 20 μm (right). **e** Quantification of hmRG mitotic somal translocation distances (*n* = 48 cells) in mice. **f** The GFP expressing hmRG (*n* = 6 independent experiments) produced both the daughter neurons that were TUJ1 positive. Time stamp, h:min. Scale bars, 50 μm (left), 20 μm (middle and right). **g** Analysis of post-time-lapse hmRG daughter cell fates in the mouse hypothalamus (*n* = 22 pairs). N: neuron, P: progenitor. **h** Time-lapse images of GFP-labeled MZ progenitor cells (arrows, *n* = 3 independent experiments) in the human hypothalamus. Arrowheads, daughter cells. Scale bars, 50 μm (left) and 20 μm (right). **i** Double labeling for EdU/BrdU in the developing mouse hypothalamus. Scale bars, 100 μm (left) and 50 μm (right). Right, high-magnification images of the outlined region, respectively, from anterior to posterior. Quantification of the percentage of EdU^+^BrdU^+^ cells among EdU^+^ cells in the VZ and MZ (*n* = 9 brain slices from 3 animals) and that in the MZ from anterior to posterior hypothalamus (*n* = 3 brain slices from 3 animals each groups). White dashed line, border of VZ and MZ. **j** Distinct centrosome positions in different progenitor cells (observed 20 hRG cells and 18 hmRG cells, respectively, from 3 independent experiments). Scale bars, 50 μm (left), 5 μm (middle), and 2 μm (right). Data are presented as mean values ± SEM in **a**, **e**, **i**. Source data are provided as a Source Data file.

We next examined the mitotic behaviors and cell fate of hRG and hmRG cells. We labeled the cells by injecting a GFP-expressing adenovirus into the third ventricle and then recorded cellular behaviors. The soma of hRG cells moved apically and divided at the surface of the third ventricle, producing one hRG cell and one hmRG cell in the mouse (Fig. 2a and Supplementary Movie 1) and human (Fig. 2b and Supplementary Movie 2) hypothalamus, both of which expressed SOX2 (Fig. 2a). The apical migration distance of the hRG cell body was 46.63 µm and the duration of mitosis was 33.78 min (Fig. 2a).

The spindle orientation was postulated to be a critical mechanism controlling the self-renewal or differentiation of daughter cells generated by progenitor cell division^31,32^. We next examined the spindle orientation in mitotic hRG cells in the VZ of the developing hypothalamus by performing PH3 immunostaining. Notably, 40.74% of mouse hRG cells divided horizontally, and 30.16% and 29.10% of the cleavage angles were oblique and vertical, respectively (Fig. 2c). Moreover, 63.89% of human hRG cells divided in horizontal and oblique orientations in the developing hypothalamus at GW12 (Supplementary Fig. 2a), suggesting that, similar to cortical RG cells, hmRG cells were generated from horizontal or oblique divisions of hRG cells in mammals.

We next asked whether hmRG cells generate neurons. We monitored hmRG cell divisions using real-time imaging and determined the daughter cell fate by immunostaining with cell type-specific markers. Human and mouse hmRG cells underwent MST before mitosis (Fig. 2d, Supplementary Fig. 2b and Supplementary Movies 3 and 4). The duration of hmRG cell body translocation was approximately 43.54 min, and the translocation distance was approximately 38.42 μm in mice (Fig. 2e). According to the cell fate analysis, we observed 13.64% hmRG (3 out of 22) divided and gave rise to two daughter neuronal cells expressing TUJ1 or NeuN (Fig. 2f, g), while 27.27% of hmRG cells (6 out of 22) divided into progenies, including one SOX2^+^ hmRG cell with a basal process and one daughter cell expressing the neuronal marker TUJ1 or NeuN (Fig. 2g and Supplementary Fig. 2c). In all, 59.09% hmRG (13 out of 22) cells’ daughter cells were both SOX2^+^ (Fig. 2g and Supplementary Fig. 2d), and some daughter cells had no basal processes (Supplementary Fig. 2d), suggesting that hmRG cells may undergo self-renewal and also generate SOX2^+^ MZ progenitors to enlarge the progenitor pool and increase the diversity of HPCs. Of the 48 mouse hmRG cell divisions, 77.08% cells divided in a horizontal cleavage plane (Supplementary Fig. 2e), which may be responsible for maintaining the hmRG population^26,32^.

Using a time-lapse analysis, we observed another type of progenitor cells in the MZ, dividing symmetrically (Fig. 2h in humans, Supplementary Fig. 2f in mice and Supplementary Movies 5, 6) to generate two daughter cells that both expressed SOX2 (Supplementary Fig. 2g). Since specific markers of MZ progenitor cells are not available, we cannot clearly estimate the population of MZ progenitors. Hence, we recorded a wide range of cell divisions in the MZ of the tuberal hypothalamus. We observed actively dividing progenitors in the hypothalamic MZ, including five hmRG cells and three MZ progenitors (Supplementary Fig. 2h and Supplementary Movie 7). We next investigated proliferation in the developing hypothalamus by performing dual-pulse labeling. An injection of 5-ethynyl-2′-deoxyuridine (EdU) was administered to pregnant mice 8 h after E12.5, and 16 h later, a 2-h bromodeoxyuridine (BrdU) pulse was administered (Fig. 2i). A small band of cells was located just outside the VZ **(**Fig. 2i), 92% of which re-entered S phase within 16 h (Fig. 2i), suggesting that HPCs (including hmRG cells and MZ progenitors) in the medial MZ exhibited high proliferative activity. In addition, the proportion of cells in the MZ that re-entered S phase decreased from the anterior to posterior hypothalamus (Fig. 2i). Taken together, our data suggest that, other than traditional hRG, hmRG and MZ progenitors also exist in the developing mammalian hypothalamus. The hmRG cells, which arose from asymmetric divisions of hRG cells, generated hypothalamic neurons and MZ progenitors.

To dissect the cellular mechanism of soma translocation, we explored the centrosome positions in hRG and hmRG cells in the mouse hypothalamus and observed that the centrosome was located in the endfeet of hRG cells at the ventricle surface during interphase (Fig. 2j). In addition, the centrosome was located in a varicosity in the basal process of hmRG cells (Fig. 2j), which may be responsible for the somal translocation before hmRG cell division.

### Early hypothalamic neurogenesis occurs in columns

**Fig. 3:**
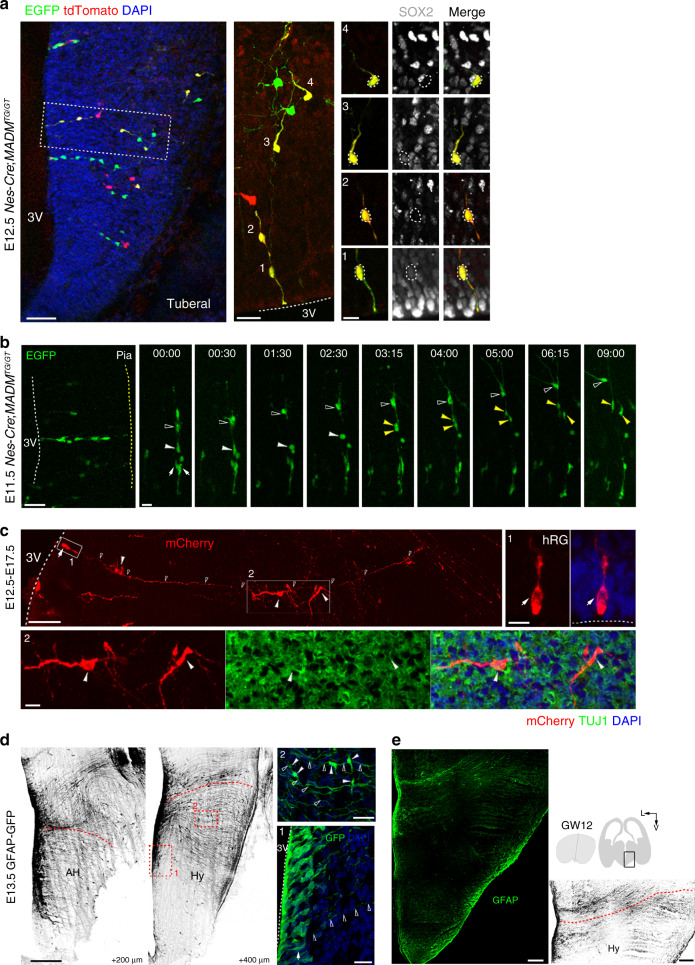
Early hypothalamic neurogenesis occurs in radial columns. **a** Labeling of radial arrays of cells (*n* = 3 independent experiments) in the developing mouse hypothalamus using MADM. A high-magnification image of the radially aligned clones is shown. The cellular composition of clones was identified by staining for the radial glial progenitor marker SOX2. Dashed lines indicate the VZ surface. White dotted lines indicate cell bodies. Right, magnified images of RG cells (area 1) and progeny (areas 2–4). Scale bars, 50 μm (left), 20 μm (middle) and 10 μm (right). **b** Time-lapse imaging of a MADM-labeled radially aligned clone (*n* = 3 independent experiments). Arrows indicate RG cells. White arrowheads indicate hmRG cells. Yellow arrowheads indicate daughter cells. Open arrowheads indicate migrating progeny. Time stamp, h:min. Scale bars, 50 μm (left) and 20 μm (right). **c** Representative mCherry-retrovirus-labeled progeny of a radial glial cell 5 days after the injection at E12.5 (*n* = 3 independent experiments). mCherry-positive hRG cell (box 1) located on the surface of the 3V. The mCherry-expressing progeny (box 2) in the MZ were positive for the neuronal marker TUJ1 (green). Arrows indicate the hRG cells. Arrowheads indicate daughter cells. Open arrowheads indicate basal processes. Dashed lines indicate the surface of the 3V. Scale bars, 50 µm (top left) and 10 µm (top right and bottom). **d** Spatial distribution of GFAP^+^ radial fibers at E13.5 (*n* = 4 independent experiments). Arrows indicate GFAP-expressing hRG cells (box 1) aligning at the surface of the third ventricle, and arrowheads indicate cells (box 2) migrating along radial fibers (open arrowheads). We changed the green GFP to black–white version to make it more visible. DNA, blue. Red dashed lines define the presumptive hypothalamus. Scale bars, 200 µm (left) and 20 µm (right). **e** GFAP expression in the developing human hypothalamus (*n* = 3 independent experiments). Scale bars, 400 µm (left and right).

We introduced *Nestin*-*Cre* lines into the chromosome 11-targeted MADM system (MADM11) to further map the neurogenic ability of progenitors in the developing mouse hypothalamus^33^. The MADM system allows dividing progenitors to restore and express either EGFP or tdTomato or mixed fluorescent markers in each of their daughter cells. We observed radial clusters of cells showing the same fluorescent markers in the embryonic hypothalamus (Fig. 3a). The clusters were radially organized and consisted of hRG cells and a number of cells with short processes arrayed along the hRG fibers (Fig. 3a). Next, we identified the cell types present in the clonal clusters and found that the bipolar hRG cells were SOX2^+^ (Fig. 3a, #1). We also observed some cells outside of the VZ that were also SOX2^+^, suggesting that these cells may be hmRG or MZ progenitors (Fig. 3a, #3). In addition to the hRG cells and MZ progenitors, radial clusters also contained cells located far away from the VZ that expressed the neuronal marker TUJ1 (Supplementary Fig. 3a, #2). Based on the statistical analysis for clone size and cellular composition of the MADM-labeled embryonic clones at E12.5, we found that, on average, individual hypothalamic clone at E12.5 was composed of 6.45 cells (Supplementary Fig. 3b), containing 20.37% hRG cells (Supplementary Fig. 3c), 22.09% hmRG cells (Supplementary Fig. 3d), and 29.37% neuronal cells (Supplementary Fig. 3e). We recorded cell division in the developing hypothalamus of MADM mice by performing time-lapse imaging of hRG and hmRG cells (Fig. 3b). One hmRG cell underwent division to produce two daughter cells (yellow arrowheads) that were incorporated into the radial column (Fig. 3b and Supplementary Movie 8). We also observed a cell with short branches (open arrowheads) that migrated radially along the hRG fibers toward the pia and then underwent tangential migration away from the clone through its leading processes (Fig. 3b and Supplementary Movie 8). In addition, we injected retroviruses expressing mCherry into the third ventricle of E12.5 mouse embryos at approximately the onset of the neurogenesis peak in the hypothalamus, and radial clusters of cells in the embryonic hypothalamus were examined (Fig. 3c). We identified 4 mCherry-labeled cells, including an RG mother cell (Fig. 3c, white arrow) and daughter TUJ1^+^ newborn neurons (Fig. 3c, white arrowheads). We also confirm that the progenitors labeled by retrovirus at embryonic stage generated neurons with high expression of NeuN in the postnatal hypothalamus (Supplementary Fig. 3f, white arrow, cells 1–4). Taken together, the cell lineage analysis using the MADM system and retrovirus tracing both indicate that hRG cells are the mother cells of hmRG cells, MZ progenitors, and neurons in the mammalian hypothalamus.

Since we observed some daughter cells lining up close to the basal fibers of RG cells, indicating the RG fibers may function as a scaffold for newborn neurons migrating to the appropriate position. Hence, we then used embryonic mice expressing GFP under control of the glial fibrillary acidic protein (GFAP) promoter (GFAP-GFP) to reveal characteristics of neural progenitors and define the topographical features of the RG basal fibers in the embryonic hypothalamus. We observed GFAP expressed in hRG cells aligned at the surface of the third ventricle in the E13.5 developing mouse hypothalamus (Fig. 3d, #1). GFAP^+^ glial fibers originating from cells located in the ventricle extended across the medial to lateral developing hypothalamus (Fig. 3d and Supplementary Fig. 3g). In addition, several GFAP^+^ cells with short processes in the MZ were aligned in the same direction as the radial fibers (Fig. 3d, #2), suggesting the association of particular orientations of radial processes with directional hypothalamic neuron migration. Similarly, the spatial pattern of GFAP^+^ fibers was also observed in the human developing hypothalamus (GW12) (Fig. 3e). Interestingly, the spatial pattern of GFAP-expressing cells was consistent with some topographical features of the NESTIN^+^ cells in the mouse hypothalamus (Supplementary Fig. 3h), suggesting that HPCs expressing specific markers may exhibit spatial and temporal heterogeneity during hypothalamic neurogenesis.

### Molecular characterization of HPCs

**Fig. 4:**
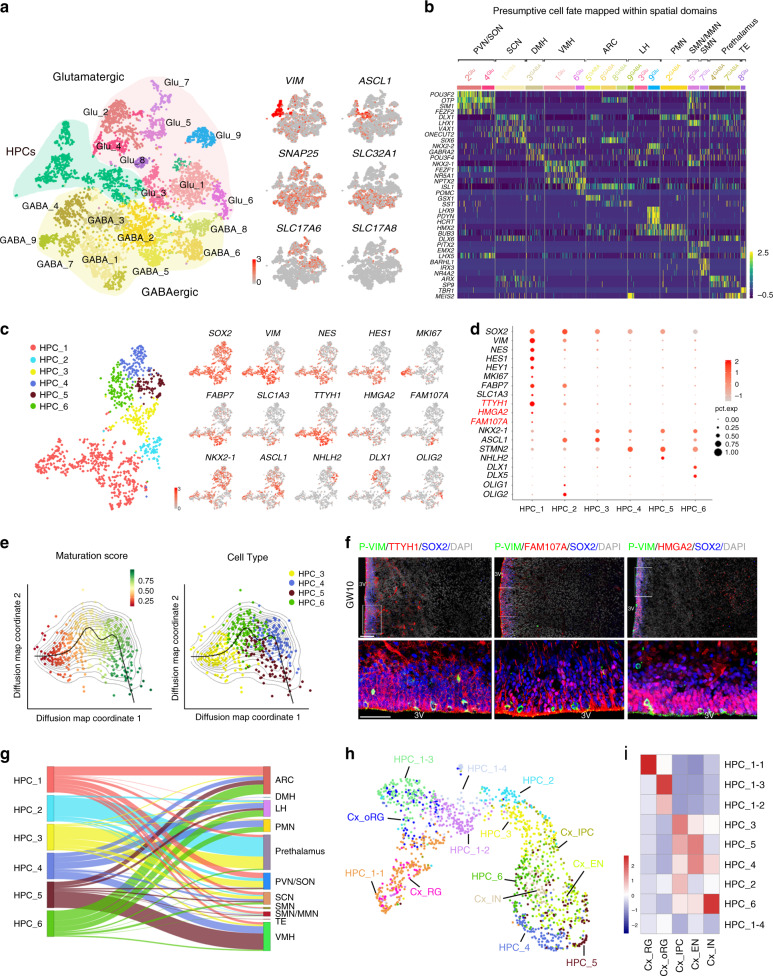
Molecular diversity of cell types in the developing human hypothalamus. **a** Visualization of major classes of cells using *t*-SNE. Different cell clusters are color coded. Right, expression of known markers in distinct cell clusters (gray, no expression; red, relative expression). HPC: hypothalamic progenitor cell, Glutamatergic: glutamatergic neuron clusters, GABAergic: GABAergic neuron clusters. **b** Heatmap showing a critical subset of region-specific genes in major hypothalamic and adjacent cell populations including the prethalamus and thalamic eminence (TE). Specific genes related to each spatial domain are highlighted on the left. The color key from purple to yellow indicates low to high gene expression, respectively. PVN/SON: paraventricular nucleus/supraoptic nucleus, SCN: suprachiasmatic nucleus, DMH: dorsomedial hypothalamus, VMH: ventromedial hypothalamus, ARC: arcuate nucleus, LH: lateral hypothalamus, PMN: premammillary nucleus, MMN: medial mammillary nucleus, SMN: supramammillary nucleus. **c** Visualization of six subtypes of hypothalamic progenitor cells using *t*-SNE (color on the left, subtypes of HPCs) with known marker expression (right: gray, no expression; red, increased relative expression). **d** Dot plot for specific markers of six subtypes of hypothalamic progenitor cells. Candidate markers are in red while known markers are in black. The color of each dot (gray, no expression; red, increased relative expression) shows average scale expression, and its size represents the percentage of cells in the subtypes. **e** A principal curve was fitted to the dominant diffusion map coordinates to order cells along a maturation trajectory. **f** Immunostaining for candidate markers including TTYH1, FAM107A, and HMGA2 with SOX2 and P-VIM, respectively (*n* = 3 independent experiments). Higher-magnification views of the boxed regions are shown at the bottom. Scale bars, 100 μm (top), 50 μm (bottom). **g** Alluvial plot showing the predicted relationship between hypothalamic progenitor clusters (left) and neuronal clusters with spatial location (right). **h** Integration of hypothalamic and cortical progenitor cells in human was visualized by UMAP, and clusters were colored differentially. Cx: cortex, EN: excitatory neuron, IN: inhibitory neurons, IPC: intermediate progenitor cell. **i** Gene expression correlation of progenitor subtypes between hypothalamus and cortex were visualized by heatmap. The scale bar indicates the Pearson correlation coefficient. Source data are supplied as a Source Data file.

To define the molecular and cellular heterogeneity of progenitor cells in the developing hypothalamus, scRNA-seq (10× Genomics Chromium) was performed in the embryonic human hypothalamus at GW10. We performed the *t*-distributed stochastic neighbor embedding (*t*-SNE) analysis described by Seurat^34^ to classify cell types in the developing human hypothalamus. Based on the differentially expressed genes (DEGs) and gene ontology (GO) of these genes, we identified cells as HPCs, nine genetic clusters of glutamatergic neurons and nine genetic clusters of GABAergic neurons (Fig. 4a, Supplementary Fig. 4a). The progenitors specifically expressed classic genes, such as *VIM*, *NES* and *ASCL1*. The neuronal clusters (including glutamatergic and GABAergic neurons) highly expressed neuronal markers *SYT1* and *SNAP25*. The presence of either *SLC17A6* or *SLC32A1* defined glutamatergic and GABAergic neurons, respectively (Fig. 4a and Supplementary Fig. 4b). To investigate the differences of neuron subtypes, we next looked at the DEGs of these cells and categorized them into distinct spatial regions by expression of transcription factors and featuring neuropeptides that are classical hypothalamus nuclei markers (Fig. 4b and Supplementary Fig. 4c).

To further underline the molecular characteristics of progenitors, we subgrouped HPCs into six clusters by *t*-SNE and analyzed DEGs in these genetic clusters (Fig. 4c). HPCs in Cluster 1 exhibited significant enrichment of known early neural progenitor genes, such as *VIM*, *NES*, and *HES1*, while cells in other clusters expressed specific genes related to neural differentiation or non-neural fate determination (Fig. 4c, d). For example, the transcription factors *OLIG1* and *OLIG2*, which control oligodendrocyte maturation, were expressed at high levels in Cluster 2 of HPCs (Fig. 4c, d), suggesting that these cells may undergo gliogenesis. HPCs of Clusters 3–6 all highly expressed *NKX2-1* and cells of Cluster 6 are also *DLX1/5*^*+*^ (Fig. 4c, d). Interestingly, Cluster 3 HPCs are *ASCL1*-high and *STMN2*-low, while Cluster 4 HPCs are *ASCL1*-low and *STMN2*-high, indicating that cells in Cluster 4 may have potentials for neural differentiation (Fig. 4c, d). In addition, Cluster 5 HPCs are selectively expressing proneural genes (such as *NHLH2*), while Cluster 6 HPCs specifically express *DLX* homeobox genes, such as *DLX1* and *DLX5*, indicating the molecular heterogeneity of HPCs. The maturation analysis of HPCs of Clusters 3–6 also suggested that HPCs of Clusters 3/6 were less mature than cells of Clusters 4/5. In addition, Cluster 4 HPCs showed relatively high maturation state than cells in Cluster 3, in consistent with *ASCL1* expression levels (Fig. 4e and Supplementary Fig. 4d). To further investigate the regulatory factors involved in differentiation potential of these progenitors, we performed the GO analysis of DEGs of these progenitor clusters and revealed that Notch signaling pathway was enriched in less-matured Clusters 3/6 (HPC_3 and HPC_6; Fig. 4e and Supplementary Fig. 4e). However, Clusters 4/5 (HPC_4 and HPC_5) with high maturation state mainly contained neuropeptide or hormone signaling pathway including oxytocin signaling and estrogen signaling, as well as synapse pathway (Supplementary Fig. 4e), implying that environment-dependent signals or factors might play roles in differentiation of HPCs.

To find RG cell markers, we focused on the cells in Cluster 1 because these cells expressed early neural progenitor genes (Fig. 4c). We found that *TTYH1*, *HMGA2*, and *FAM107A* were highly expressed in Cluster 1 cells (Fig. 4d, red labels). Next, we performed immunostaining of the GW10 human hypothalamus to further validate these candidates. TTYH1-, FAM107A-, or HMGA2-expressing cells residing in the VZ were positive for SOX2 and P-VIM (Fig. 4f). A few TTYH1^+^SOX2^+^ cells were also observed in the medial MZ, location of the hmRG and MZ progenitors (Fig. 4f). FAM107A^+^SOX2^+^ cells exhibited radial fibers extending basally toward the pial surface, suggesting that these cells are hRG and hmRG cells (Fig. 4f). We also observed some HMGA2-expressing cells lacking SOX2 that were sparsely distributed in the lateral MZ, indicating that they possibly were postmitotic cells (Fig. 4f). To further reveal the potential cell fate of HPCs, we performed integrative analysis of progenitor cells and neurons (Supplementary Fig. 4f) and plotted the potential developmental relationship among them (Fig. 4g). According to the transcriptome similarity, the cellular distributions by UMAP representation could indicate developmental relationship among integrated genetic clusters^35^. We found that Cluster 1 HPCs characterized as RG populations were predicted to generate diverse neuronal progenies, and Cluster 5 HPCs were mainly mapped into VMH cell populations (Fig. 4g and Supplementary Fig. 4f).

Cluster 1 HPCs expressed classic RG markers, such as *NES*, *VIM*, *FABP7*, and *SLC1A3* (Fig. 4c, d); we next asked whether hRG and hmRG could be distinguished. So we subclassified these progenitors (HPC_1) into four subsets (Supplementary Fig. 4g, h). We found that both subclusters (HPC_1-1 and HPC_1-2) were possibly hRG cells because they highly expressed early well-known transcription factors of HPCs in VZ (*SHH*, *RAX*) and components of BMP signaling pathway regulating self-renewal and hypothalamic patterning (Supplementary Fig. 4h). The HPC_1–3 were likely to be hmRG cells that were characterized by the presence of cell-surface proteins (Supplementary Fig. 4h), such as *VCAM1*, *FABP7*, and *FAM107A* and enriched in elements of Wnt signaling pathway (Supplementary Fig. 4h). In addition, cells of HPC_1–4 displayed high expression of extracellular matrix molecule, indicative of presumptive neuroepithelial cells (Supplementary Fig. 4h).

To study the differences between hypothalamic and cortical neural progenitors, we integrated the scRNA-seq data of human HPCs with embryonic cortical progenitors^36^ and calculated the gene expression correlations between the hypothalamic and cortical progenitor (Fig. 4h, i). The subset of HPC_1-1 was highly correlated with the cortical RG cells (Cx_RG) and the cells of HPC_1–3 were similar to cortical oRG populations (Cx_oRG) (Fig. 4i). In addition, other clusters of HPC (Cluster 3/4/5/6 HPCs) that showed distinctive maturation state were highly correlated with the cortical intermediate progenitor cells (IPCs) and immature neurons, respectively (Fig. 4i). Despite of multiple similar features of morphology and cell division behavior shared by the hmRG in the hypothalamus and oRG in the cortex, these two types of progenitor cells still showed molecular differences (Supplementary Fig. 4i).

**Fig. 5:**
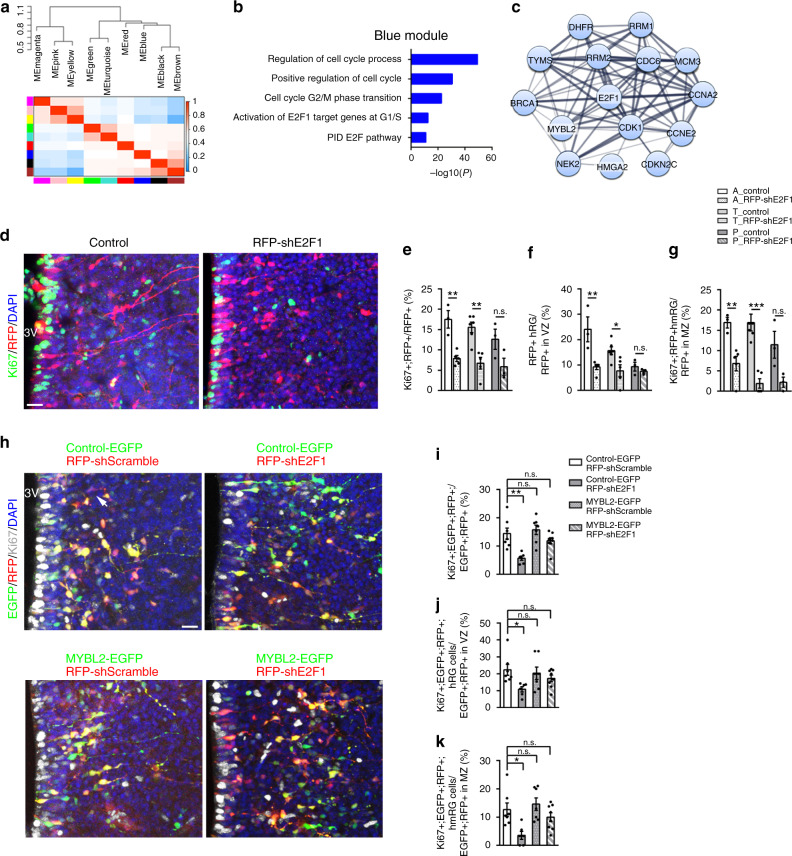
Molecular signature of neural progenitor cells in the developing hypothalamus. **a** Eigen gene heatmap showing the relationships among the modules across hypothalamic progenitor population. **b** Major gene ontology terms or pathways associated with the blue modules. **c** Functional network of genes/proteins involved in regulation of cell cycle. The network was generated with the STRING tool. **d** The knockdown of E2F1 decreased proliferating progenitor in mice hypothalamus (*n* = 3 independent experiments). Representative images of E14.5 hypothalamic sections electroporated with shControl (red) and shE2F1 (red) plasmids at E12.5 and immunostained with Ki67. Scale bar, 50 μm. **e**–**g** Quantification for the percentage of Ki67^+^ cells among RFP^+^ cells (**e**), Ki67^+^ hRG cells among RFP^+^ cells in hypothalamic VZ (**f**) and Ki67^+^ hmRG cells among RFP^+^ cells in hypothalamic MZ (**g**), respectively in the anterior–posterior axes. A: anterior, T: tuberal, P: posterior (*n* = 3 independent experiments); *p*^A_control vs. A_RFP-shE2F1^ = 0.002 (**e**), *p*^T_control vs. T_RFP-shE2F1^ = 0.003 (**e**), *p*^P_control vs. P_RFP-shE2F1^ = 0.1056 (**e**); *p*^A_control vs. A_RFP-shE2F1^ = 0.0084 (**f**), *p*^T_control vs. T_RFP-shE2F1^ = 0.0176 (**f**), *p*^P_control vs. P_RFP-shE2F1^ = 0.3729 (**f**); *p*^A_control vs. A_RFP-shE2F1^ = 0.008 (**g**), *p*^T_control vs. T_RFP-shE2F1^ = 0.0002 (**g**), *p*^P_control vs. P_RFP-shE2F1^ = 0.0579 (**g**); *p < 0.05, **p < 0.01, ***p < 0.001, two-tailed Student’s *t* test. **h** Overexpression of MYBL2 rescues the hypothalamic progenitor decrease by E2F1 knockdown in mice (*n* = 3 independent experiments). Co-expression of shE2F1-RFP and MYBL2-EGFP using electroporation at E12.5 mice. Samples of the E14.5 hypothalamus are immunostained by Ki67. Scale bar, 50 μm. **i**–**k** Quantification for the percentage of Ki67^+^ cells among electroporated EGFP^+^RFP^+^ cells. *n* = 7 brain slices from 3 animals in Control-EGFP^+^ shScramble, 6 brain slices from 3 animals in Control-EGFP^+^ shE2F1, 7 brain slices from 3 animals in MYBL2-EGFP^+^ shScramble, and 8 brain slices from 4 animals in MYBL2-EGFP^+^ shE2F1. One-way ANOVA and Tukey’s test to compare data sets. n.s.: not significant. *p < 0.05, **p < 0.01. Data are presented as mean values ± SEM. Source data are supplied as a Source Data file.

To further define the molecular features of HPCs, we performed weighted gene correlation network analysis (WGCNA)^37^ of variable genes of HPCs. Nine gene modules were identified (Fig. 5a and Supplementary Fig. 5a). These modules were next examined in combination with functional enrichment analysis to search the characteristic biological pathways. Genes associated with the cell adhesion, such as *TTYH1* and *FAM107A*, were presented in brown modules (Supplementary Fig. 5b). Notably, the GO terms of genes in the blue module are associated with regulation of cell cycle and E2F pathway (Fig. 5b). The gene-network analysis further revealed a hierarchical organization of highly connected genes involved in the process of cell cycle and E2F pathway, including *HMGA2*, *E2F1*, *MYBL2*, and *CDK1* (Fig. 5c). To investigate whether the transcription factor E2F1 regulate progenitor cells in the developing hypothalamus, we electroporated E2F1 short hairpin RNA (shRNA) (shE2F1) into the mouse hypothalamus via in utero electroporation (IUE) at E12.5 (Supplementary Fig. 5c). Two days after transfection, the proliferation ability of electroporated hypothalamic cells (RFP^+^) was analyzed by Ki67 labeling (Fig. 5d). With E2F1 knockdown in hypothalamic cells, proliferating progenitor cells (Ki67^+^RFP^+^) significantly decreased compared with that of the shControl-RFP-electroporated samples (Fig. 5e). We also observed that the suppression of E2F1 expression resulted in decreasing the percentage of hRG cells in VZ and hmRG cells with basal processes in MZ (Fig. 5f, g), suggesting that E2F1 plays a role in HPC proliferation. However, we found some differences in the anterior–posterior position within hypothalamus (Fig. 5e–g). The tuberal region was the most affected, but the changes of posterior regions were not statistically significant, possibly due to only very few progenitors observed in posterior hypothalamus (Fig. 1e and Supplementary Fig. 1e).

Given that MYBL2 was also in the gene-network analysis, to determine whether E2F1 regulated hypothalamic cell proliferation together with MYBL2, we examined the effect of increasing MYBL2 levels in E2F1 knockdown cells. When shE2F1 was co-electroporated with an *MYBL2*-expressing vector (MYBL2-EGFP), the decline of proliferating progenitor induced by knockdown of E2F1 was partially rescued (Fig. 5h–k). Since we observed the obvious reduction of progenitors in tuberal region, we only chose the tuberal region for quantification analysis here. These results indicate that E2F1, with its downstream factor MYBL2, promotes progenitor proliferation in the developing hypothalamus.

### Regional transcription factor expression in the developing hypothalamus

**Fig. 6:**
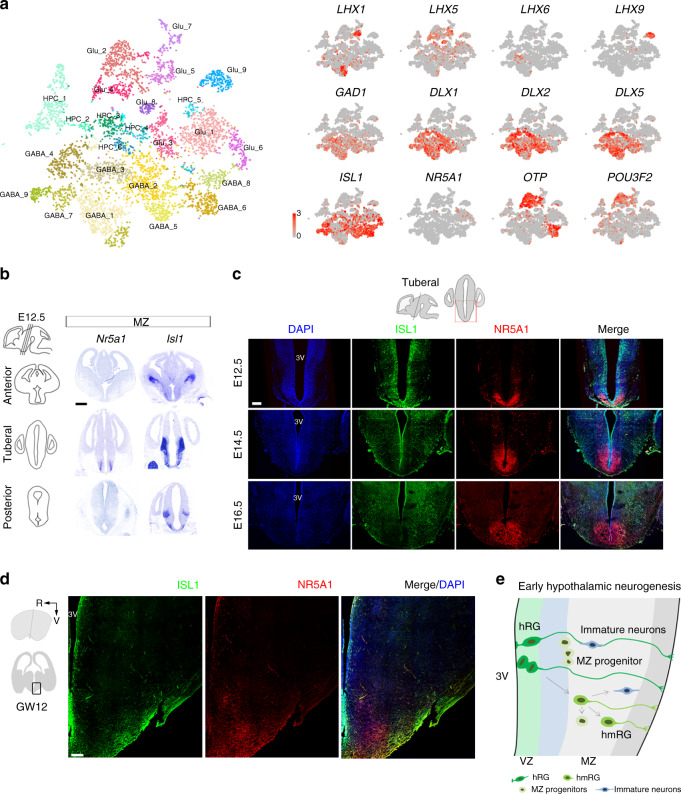
Specific regional transcription factors in the developing mammalian hypothalamus. **a***t*-SNE plot displaying well-known markers for neuron subpopulations (gray, no expression; red, relative expression). **b** In situ hybridization of *Isl1* and *Nr5a1* at E12.5 hypothalamus. Transcript expression is observed in the anterior, tuberal, and posterior regions. Scale bars, 500 μm. **c** Immunostaining for ISL1 (green) and NR5A1 (red) in the mouse hypothalamus at E12.5, E14.5, and E16.5 (*n* = 3 independent experiments for each development age). DAPI, blue. Scale bar, 200 μm. **d** Immunostaining for ISL1 (green) and NR5A1 (red) in the human hypothalamus at GW12 (*n* = 3 independent experiments). R: rostral, V: ventral. Scale bars, 200 μm. **e** Proposed model for early hypothalamic neurogenesis.

The adult hypothalamus consists of diverse cell types that are essential for regulating physiological and behavioral homeostasis^2^. A definition of the cell composition and the identification of cell-type-specific transcription factors in the hypothalamus are essential to understand its functions. Based on the results of the scRNA-seq analysis, we identified nine glutamatergic neuron subtypes (Glu1–Glu9) (Fig. 4a and Supplementary Fig. 5d, e) and nine GABAergic neuron subtypes (GABA1-GABA9) (Fig. 4a and Supplementary Fig. 5f, g). A number of well-known transcription factors were expressed at high levels in specific neuron subtypes in the developing human hypothalamus, including *ISL1*, *NR5A1*, *OTP*, *POU3F2*, the *DLX* gene family, and the *LHX* gene family (Fig. 6a). *DLX* family genes (*DLX1*, *DLX2*, and *DLX5*) were generally expressed in GABAergic neurons, except for GABA6/8 neurons (Fig. 6a and Supplementary Fig. 5f), while the *LHX* genes appeared to define specific subpopulations of hypothalamic neurons, as *LHX1* was expressed in Glu5 and GABA1 (Fig. 6a and Supplementary Fig. 5d, f), *LHX5* was expressed in Glu4/5/8 (Fig. 6a and Supplementary Fig. 5d, e), *LHX6* was expressed in GABA3 (Fig. 6a and Supplementary Fig. 5f), and *LHX9* was expressed in Glu9 (Fig. 6a and Supplementary Fig. 5d, e).

Hypothalamic development is regulated by a variety of transcription factors that are evolutionary conserved across species^1,3,38,39^. Therefore, we investigated whether the transcriptional characteristics and timing of neuronal or nuclear differentiation are similar between the rodent and human hypothalamus. The *ISL1* that plays crucial roles in cell fate decisions and nuclear development of developing hypothalamus, including ARC and VMH^15,16,40^. We found that *ISL1* was expressed in Glu3/6 neurons and all GABAergic neurons (Fig. 6a and Supplementary Fig. 5d–g). *NR5A1* is essential for the terminal differentiation and maintenance of the developing VMH cellular population^40,41^, which was selective expression in glutamatergic neurons (Glu1/6; Fig. 6a and Supplementary Fig. 5d). *OTP* and *POU3F2* were mainly expressed at high levels in cells of Clusters Glu2/4 (Fig. 6a and Supplementary Fig. 5d, e) and are required for neuron differentiation in the anterior hypothalamus^2,42^.

We next performed in situ hybridization of *Nr5a1* and *Isl1* in the developing mouse hypothalamus at E12.5 to further analyze the evolutionary conservation of the mechanism regulating the development of the mammalian hypothalamus. *Nr5a1* was distinctly expressed in the ventromedial hypothalamic MZ, the presumptive VMH nucleus. *Isl1* exhibited significant differential expression from the rostral to caudal hypothalamus across the dorsal–ventral mantle zone, which includes the presumptive VMH (Fig. 6b and Supplementary Fig. 6a). We also performed immunostaining for ISL1 and NR5A1 to identify the expression pattern of the marker proteins in the developing mouse and human hypothalamus and to further confirm the structural homology between the mouse and the human nuclei. The spatially different expression of these two proteins in the MZ was consistent with the aforementioned in situ hybridization data at E12.5 (Fig. 6c). The regional expression of these markers became more distinct as the hypothalamus developed. At E14.5, a later period of neurogenesis in the mouse, ISL1^+^ cells accumulated in the ventro-lateral mantle domain, with scattered labeling in the medial region. At E16.5, the end of neurogenesis in the mouse hypothalamus, distinct ISL1^+^ cells mainly occupied the MZ (Fig. 6c and Supplementary Fig. 6b). In comparison, NR5A1 was particularly strongly expressed in the presumptive VMH nuclei throughout hypothalamic neurogenesis from E12.5 to E16.5 (Fig. 6c and Supplementary Fig. 6c). Meanwhile, the spatial expression pattern observed later in the neurogenesis of the mouse hypothalamus was also detected in the fetal human hypothalamus at GW12 (Fig. 6d), suggesting a conserved pattern by which NR5A1 and ISL1 specify neuron subpopulations and subregions in the developing mammalian hypothalamus. To further evaluate similarity and difference between human and mouse, we integrated our scRNA-seq data (only cells related to ARC development) with that of the mouse embryonic ARC^43^. We subset human and mouse ARC neuronal cells into 7 groups each (Supplementary Fig. 6d). We further calculated the gene expression correlation between the ARC neuronal subclusters in humans and mice. Some neuronal subclusters, including GHRH neurons, SST neurons, POMC neurons, and LHX1-expressing neurons, showed high conservation in different species (Supplementary Fig. 6e). However, h-CARTPT/CBLN4 neurons in human displayed a weak correlation with mouse subclusters (Supplementary Fig. 6e). Moreover, these data indicated that the evolution conservative hypothalamic cell types may also show some differences in gene expression patterns at single-cell level during development.

## Discussion

The structure and function of various hypothalamic nuclei have been well studied for many years^2,7,44–46^. However, many important aspects of hypothalamic development remain unaddressed, particularly the progenitors responsible for neurogenesis in the hypothalamus. Our findings reveal the cellular and molecular features of hypothalamic neural progenitors and progenitor zones. Here we proposed a model in which hRG and additional basal progenitor classes, including hmRG and MZ progenitor cells that are mainly located in the VZ and MZ of the third ventricle, functioned as hypothalamic neural progenitor cells and were responsible for neurogenesis (Fig. 6e). During early neurogenesis, the basal processes of hRG and hmRG cells extend to the pial surface and potentially serve as a scaffold for daughter cells migrating to their final positions. In addition, the scRNA-seq analysis revealed the transcriptional heterogeneity of neural progenitor cells and regulation of early neural fate determination in the developing mammalian hypothalamus. Together, our studies elaborate the cellular and molecular properties of hypothalamic neural progenitor cells, and thus our findings provide new insights into the mechanism of hypothalamic neurogenesis.

RG and oRG/basal RG (bRG) are well-known neural progenitor cells that are responsible for producing projection neurons in the rodent and primate neocortex^25,26,28,47,48^. As early as 1980, Levitt and Rakic observed RG cells in the VZ of the third ventricle in E45 rhesus monkey embryos using GFAP labeling^49^. Similar to this observation, we identified hRG cells in the developing human hypothalamus at GW11. In addition to classic hRG cells, we also observed RG-like cells (hmRG) that only extend basal processes and lack contact with the ventricular surface. The morphology of these cells was similar to oRG/bRG cells in the developing neocortex. Retrovirus labeling or the use of the MADM system revealed that hRG cells were not only the mothers of neurons but also the ancestors of hmRG cells. Interestingly, HPCs share the same cell division behaviors as cortical progenitors, as hRG and hmRG cells underwent INM and MST, respectively, which was determined from the location of the centrosomes in these cells. In both the human and mouse cortex, RGs have been shown to produce oRGs via horizontal or oblique divisions^32,50,51^. According to an analysis of images of fixed mouse and human hypothalamus sections, the majority of VZ divisions during neurogenesis display non-vertical cleavage angles, suggesting that one daughter may adopt the hmRG cell fate after hRG division. In addition, our time-lapse data show that hRG divisions produce basal daughters with a hmRG morphology during hypothalamic neurogenesis.

In mammals, oRG cells exhibit the self-renewal capability and directly^25^ or indirectly generate neurons through the production of IPCs^26^, which undergo transit amplifying divisions to increase neuron production during periods of neocortical neurogenesis. Similar to oRG cells^25^, a subset of hmRG cells in the mouse hypothalamus were responsible for neurogenesis by undergoing asymmetric division to directly generate neurons. Based on our cell fate analysis, the apical daughter cells were neurons, but the basal daughter acquired an hmRG morphology and expressed SOX2. Interestingly, in the majority of hmRG cell divisions we monitored, both hmRG daughters expressed SOX2, indicative of their progenitor fate. However, further studies are required to determine whether these mouse hmRG cells exhibit a similar proliferative potential to human hmRG cells. These results suggested diversity or heterogeneity within the mouse hypothalamic hmRG populations in terms of daughter cell fate. Interestingly, we observed MZ progenitors that symmetrically divided and exhibited a very active cell cycle. However, the daughter cells of these MZ progenitors expressed SOX2 but not neural markers. We hypothesized that these MZ progenitors may contribute to gliogenesis in the developing hypothalamus.

A remarkable feature of the neocortex is the inside-out arrangement of projection neurons, which is strongly correlated with the directions of basal processes from RG and oRG cells in mammals^25,26,28,52,53^. Although we also observed that hRG and hmRG fibers extended in a fan formation from the VZ and MZ to the pial surface, hypothalamic neuronal development is known to occur in an outside-first manner by organizing the nuclei but a layer-like structure is not produced^45,46^. GFAP^+^ radial fibers extended from the third ventricle to the surface of the brain in a curved manner, presumably to form specific hypothalamic regions, consistent with a previous study^49,54^. These fibers may serve as guides for migrating neurons. During the very early embryonic stage, hRG cells and their progeny are organized radially along a mediolateral axis. Due to the limited expansion of the anterior–posterior axis, the scaffold fibers of hRG or hmRG cells may exhibit a relatively radial position during early development. Previous studies have reported important roles for cell adhesion molecules in regulating cell migration. For example, Cadherin-11^55^, PCDHα^56^, and PCDH9^57^ are expressed in the developing mammalian hypothalamus. Consistent with these findings, ZO-1, β-catenin, and N-cadherin were broadly expressed in the developing mouse hypothalamus. In addition, Tobet and colleagues revealed that GABA receptor signaling is involved in cell motility in the VMH^58,59^, suggesting that the migration and orientation of newborn neurons may be regulated by both intrinsic and extrinsic signals that play critical roles in determining the final location of neurons. Hence, the column patterns may be disrupted when nuclei start to form.

We have identified neural progenitor cells with diverse cellular morphologies in the mammalian hypothalamus. However, the molecular mechanisms underlying progenitor heterogeneity remain elusive. Here we revealed the cell-type-specific transcriptome of the human hypothalamus using scRNA-seq. We showed remarkably diverse transcription factor expression patterns within progenitor subtypes, which were characterized by the combinatorial or specific expression of classic progenitor markers (*VIM*, *NES*, *FABP7*, and *SLC1A3*) and proneural transcription factors (*ASCL1*, *NHLH2*, and *DLX1*). Further analyses indicated that the hRG and hmRG exhibited molecular differences. In addition, cortical and hypothalamic RG were similar while cortical oRG and hypothalamic hmRG were more likely to be correlated. The scRNA-seq analyses indicated that the glutamatergic and GABAergic neurons were differentiated and subgroups could be detected in the GW10 human hypothalamus. We tried to predict the presumptive spatial distribution of these cells based on gene expression levels of regional- or cell-type-specific markers typically used for adult nuclei identification^20,21,24,60,61^. One thing we have to say is that the hypothalamus neurons we have analyzed were immature. This spatial prediction may not be very accurate but still could give us some useful information and indicate the potential cell fate.

In addition, we identified several new neural progenitor markers, such as *FAM107A*, *TTYH1*, and *HMGA2*, playing roles in cell proliferation. Additional gene-network enrichment analysis among HPC population indicated that HMGA2 is in the network of E2F1 pathway. The E2F pathway has been shown to play important roles in promoting cell cycle progression^62,63^. By both scRNA-seq data analysis and in vivo experiments, we consistently identified that E2F1 pathway plays an essential role in regulating the proliferation of HPC by acting on the downstream MYBL2.

The hypothalamus is an evolutionarily conserved part of the brain because it acts as an integrator to regulate and coordinate basic functions necessary for the survival of the individual^2–4^. The peak birthdate of hypothalamic nuclei is E13–E15 in rats^45,64^ and E12–E14 in mice^46,65^. Rakic and van Erdenburg observed the development of the hypothalamus between the E27 and E48 in the macaque monkey^66^. Consistent with previous studies, although the cytoarchitecture of the primate hypothalamus is not exactly the same as the rodent hypothalamus and neurogenesis progression is initiated at an earlier stage of gestation in primates than in rodents^45,46,66,67^, some of the basic neurogenic characteristics were conserved in our study. We identified hRG and hmRG cells in both the mouse and human hypothalamus, and these cells showed similar cellular and molecular properties. Moreover, some key transcription factors that specify hypothalamic neuron subpopulations^1^ were conserved between mouse and human. In summary, our findings fill the gaps in our understanding of the cellular and molecular mechanisms underlying the development of the mammalian hypothalamus. Further studies examining how circuits are constructed at different development stages will provide researchers new opportunities to investigate the function of the hypothalamus and its association with the related diseases.

## Methods

### Mice

All animal procedures used in this study were performed in accordance with the protocol approved by the Institutional Animal Care and Use Committee of the Institute of Biophysics, Chinese Academy of Sciences. All mice had free access to food and water and were housed in the institutional animal care facility (specific pathogen free, at temperature of 21 °C, relative humidity of 60%) with a 12-h light–dark schedule. All efforts were made to minimize the number of animals used and their suffering.

Wild-type CD-1 mice purchased from Charles River Laboratories in China (Vital river, Beijing, China) were used for experiments, including DiI labeling, time-lapse imaging, IUE, retrovirus infection, in situ hybridization, and immunofluorescence.

Nestin-Cre mice (B6.Cg-Tg(Nes-cre)1Kln/J No. 003771) were purchased from Jackson laboratory. GFAP-GFP transgenic mice were constructed and kept in the FVB background. *MADM11*^GT^ (JAX Stock No. 013749) and *MADM11*^TG^ (JAX Stock No. 013751) were utilized. For MADM labeling, *Nes*-*Cre*;*MADM11*^GT/GT^ mice were crossed with *MADM11*^TG/TG^ mice, and the time of pregnancy was determined by the presence of the vaginal plug. All mice had free access to food and water and were housed in the institutional animal care facility with a 12-h light–dark schedule. All the subjects were not involved in any previous procedures.

### Human subjects

The de-identified human tissue collection and research protocols were approved by the Reproductive Study Ethics Committee of Beijing Anzhen Hospital and the institutional review board (ethics committee) of the Institute of Biophysics. The informed consent was designed as recommended by the ISSCR guidelines. The fetal tissue samples were collected after the donor patients signing informed consent document followed the legal and institutional ethical regulations of Beijing Anzhen Hospital. All the protocols were in compliance with the Interim Measures for the Administration of Human Genetic Resources, administered by the Ministry of Science and Technology of China. The gestational age was measured in weeks from the first day of the woman’s last menstrual cycle to the sample collecting date.

### DiI labeling

Embryonic mouse brains or human brain slices were fixed with 1.5% paraformaldehyde (PFA) in phosphate-buffered saline (PBS). DiI crystal (1,1′-dioctadecyl-3,3,3′,3′-tetramethylin-docarbocyanine perchlorate; D-282, Molecular Probes) was applied to the hypothalamic pia surface of the fixed mouse brains (E13.5 or E15.5) and human slices (GW11), which was allowed to diffuse throughout the tissue in PBS for 2–7 days at room temperature (RT). Labeled brains or slices were vibratome-sectioned for imaging analysis.

### Plasmids and IUE

For the construction of vectors encoding small interference RNA targeting E2F1 (shE2F1), E2F1 shRNA sequences were cloned into pLL3.7-Tdtomato. To construct gene expression plasmid, MYBL2 sequence was cloned into the pEGFP-C1 vector.

Electroporation was carried out with minor modifications as previously reported^68^. Briefly, pregnant mice (E12.5) were deeply anesthetized, and the uterine horn was exposed by midline laparotomy. One microliter of plasmid solution (1–2 µg µl^−1^) with fast green (0.1 mg ml^−1^, Sigma) was manually microinjected through the uterus wall into the third ventricle. The electroporation was performed according to the following procedure: five porting pulses of 40 mV with 50-ms pulse width and 950-ms pulse interval through two electrode paddles (9 mm) placed on either side of the head (ECM830, BTX). After the injection and electroporation were completed, the uterine horn was return to the peritoneal cavity. The animals were placed in incubator at 28 °C to recover.

### Retroviral in utero infection

Uterine horns of E12.5 gestation-stage pregnant CD-1 mouse were exposed in a clean environment. mCherry-expressing retrovirus (~0.5 µl) with fast green (0.1 mg ml^−1^, Sigma) was injected into the third ventricle with a beveled, calibrated glass micropipette (Drummond Scientific). Throughout these surgical procedures, the uterus was constantly bathed with warm PBS (pH 7.4). After injection, the uterine horns were replaced and the wound was surgically closed.

### Hypothalamic slice culture and time-lapse imaging

Brain slices were prepared for time-lapse imaging from mouse E12.5 and human GW7. The human tissue was collected within 2 h in ice-chilled artificial cerebrospinal fluid (ACSF). Briefly, the sample tissue was embedded in 3% low-melting-temperature agarose in ice-chilled ACSF and vibratome-sectioned at 300 μm in ice-chilled ACSF containing (in mM) 125 NaCl, 2.5 KCl, 1 MgCl_2_, 2 CaCl_2_, 1.25 NaH_2_PO_4_, 25 NaHCO_3_, 25 D-(+)-glucose and bubbled with 95% O_2_/5% CO_2_. Brain slices were transferred and suspended on slice culture inserts (Millipore) with culture medium in culture well plates. Cultures were incubated at 37 °C with constant 5% CO_2_ supply. For time-lapse imaging, brain slices were cultured in medium containing 66% basal medium eagle, 25% Hanks balanced salt solution, 5% fetal bovine serum, 1% N-2 medium, 1% penicillin/streptomycin/glutamine (all Invitrogen), and 0.66% D-(+)-glucose (Sigma). Cytomegalovirus (CMV)-GFP adenovirus (2 × 10^3^ colony-forming units) was microinjected focally into the third ventricle surface along the slice to achieve sparse labeling. Approximately 24 h after infection of hypothalamic slices with a CMV-GFP adenovirus (Adeno-GFP), we began to record the cell divisions using inverted laser scanning microscope (Olympus Fluoview FV1000) with a ×10 air objective lens (zoom 2) every 15–30 min for about 72 h. The time 0:00 was defined as when the characterized morphology of progenitor cells clearly displayed before cell division. Maximum intensity projections of the collected stacks were compiled and generated into movies and analyzed using the ImageJ software.

The cleavage plane angles were calculated by determining the angle between cytokinesis and the ventricular surface. MST length was measured as the distance between the site of cytokinesis and the center of the soma before the onset of translocation.

### Categorization of cleavage plane angles

Mitotic cells were identified based on PH3 immunofluorescence. Cleavage planes have been classified according to their orientation relative to the apical ventricle surface. Cleavage planes were classified into three groups: an orientation at a 0–30°, 30–60°, and 60–90° angles relative to the ventricle surface is referred to as horizontal, oblique, and vertical cleavages, respectively, which were measured with ImageJ.

### EdU/BrdU double pulsing

The CD-1 pregnant female mice were injected with 50 mg kg^−1^ EdU (Sigma) at E12.5 and 50 mg kg^−1^ BrdU (Sigma) 16 h later. After 2 h, pregnant females were sacrificed, and the brains were dissected and fixed immediately in fresh-cold 4% PFA in PBS at 4 °C overnight, followed by sequent dehydration in 20 and 30% sucrose in PBS at 4 °C. Slices were stained using EdU Click-iT (Thermo Scientific) and standard procedures.

### Tissue dissection, single-cell dissociation, and library construction

Fetal hypothalamus samples were collected in ice-cold ACSF. The hypothalamus was vibratome-sliced coronally at 600 μm in ice-chilled ACSF. Hypothalamic tissues were then dissected under dissection microscope and subjected to tissue dissociation. The hypothalamus tissues were enzymatically digested using a papain-based dissociation protocol previously reported^69^. In short, tissues samples were dissociated into single-cell suspension by digestion buffer (1 mg ml^−1^ papain in hibernate E medium, Sigma). The cell suspensions were vortexed and incubated on the thermo cycler (500 *g*) at 37 °C for 15–20 min.

Single-cell suspensions were prepared in PBS containing 0.04% bovine serum albumin at an optimal concentration and used for scRNA-seq experiment (10× Genomics).

cDNA libraries were generated using the Single Cell 3′ Reagent Kits provided by 10× Genomics, following the manufacturer’s instructions. Briefly, single-cell suspensions were loaded on Chromium instrument (10× Genomics) to get single-cell barcoded gel beads in emulsion and followed by amplification, purification, and assessment for quality control. The library sequencing was further performed on Illumina platform with 150 bp pair-end reads.

### scRNA-seq data preprocessing with chromium system

We used Cell Ranger (v2.0.1) to demultiplex FASTQ files and align reads to the hg19 human genome. We excluded poor quality cells after the gene-cell data matrix was generated by the cell ranger software using the Seurat package (v2.3.4) in Bioconductor. Only cells that expressed >800 genes were considered, and only genes expressed in at least 7 single cells (0.1% of the data) were included for further analysis. Cells that expressed hemoglobin genes (*HBA1*, *HBA2*, *HBB*, *HBD*, *HBE1*, *HBG1*, *HBG2*, *HBM*, *HBQ1*, and *HBZ*) were also removed. The data were natural log transformed and normalized for scaling the sequencing depth to a total of 1e4 molecules per cell, followed by regressing-out the number of UMI using Seurat package.

### Dimensionality reduction and cluster analysis

Seurat R Package (v2.3.4) was applied for further scRNA-seq data analysis. Highly variable genes were obtained as genes with dispersion >1 and normalized expression between 0.0125 and 3, followed by principal component analysis (PCA) on 799 most variable genes. The top 15 principal components (PCs) were used to perform *t*-SNE clustering. Identification of significant clusters was performed using FindClusters function with a resolution of 1.5. Cluster cells were then classified according to the combination of specific gene markers and GO analysis. DEGs among clusters were defined using the Seurat’s function FindAllMarkers (thresh.use = 0.25, min.pct = 0.25, Wilcoxon test). Functional annotations of DEGs were obtained using DAVID 6.7 (https://david-d.ncifcrf.gov/) and Metascape (http://metascape.org/gp/index.html#/main/step1).

### WGCNA analysis

We performed co-expression network analysis using the R WGCNA package^37^ (WGCNA version 1.66, https://cran.r-project.org/src/contrib/Archive/WGCNA/). Highly variable genes of HPC population were detected by FindVariableGenes in Seurat. Two thousand five hundred and fourteen variable genes were supplied to WGCNA. Gene modules were examined by dynamic hybrid cut. The pathway/ontology enrichment analysis of module genes performed using the Metascape (http://metascape.org). We chose correlated genes involved in E2F pathway and cell cycle process in blue module and then performed the construction of gene co-expression networks with STRING database (https://string-db.org/).

### Identification of maturation trajectory

To define a maturation trajectory, we first performed dimensionality reduction by diffusion maps and then fit a principal curve (R package princurve version 2.1.4). We assign the direction of the curve based on correlation of the gene expression value of ASCL1 with maturation.

### Integration of HPCs and distinct regional neurons

To explore possible links between of progenitor cell clusters and spatial neuronal clusters by our scRNA-seq data, we combined and identified the integration of anchor genes between HPC subtypes and regional neuron subpopulations with Seurat v3 function FindIntegrationAnchors. Cell clustering and dimensionality reduction were performed with function FindClusters and RunUMAP. The UMAP-based transcriptome analysis was performed for visualizing hypothalamic cells according to single-cell transcriptional similarity. The closer physical distance of cell points in the UMAP indicates high similarity of genetic pattern among them.

### Integration of hypothalamus and cortex dataset

To evaluate differentiation homology and heterogeneity among different brain regions, we integrated HPC cells with human cortical database that was downloaded from previous published work^36^ by aligning them using Seurat v3 function FindIntegrationAnchors. Using Seurat’s IntegrateData function, samples were combined into one object. PCA was used, and the first 6 PCs were summarized further using UMAP dimensionality reduction.

### Integration of human and mouse dataset

To investigate the difference in hypothalamic cells between human and mouse, we extracted presumptive ARC neurons in human and classic embryonic ARC neurons from recently published clusters^43^ and then performed the integration analysis using Seurat v3.

The two datasets were merged utilizing the FindIntegrationAnchors function. The IntegratData function for batch correction for all cells were ran for enabling these datasets to be jointly analyzed. Clusters were visualized by UMAP with Seurat’s RunUMAP function.

### Gene expression correlation analysis

The Pearson correlation coefficient across hypothalamus and cortex progenitor cells or human and mice ARC neurons were calculated with shared variable genes in both datasets. The resulted correlation matrices were visualized with the R software and pheatmap package version 1.0.12.

### cRNA probe synthesis and in situ hybridization

For in situ hybridization, all operations were performed under RNase-free conditions. Related primer sequences were described as the following:

*Nr5a1* (Forward: 5′-CGCGGATCCCCCAAGAGTTAGTGCTCCAGTT-3′, Reverse: 5′-CCGGAATTCATGTTGGCTACACCAGACTCCT-3′),

*Isl1* (Forward: 5′-CCCAAGCTTGAAATGTGCGGAGTGTAATCAG-3′, Reverse: 5′-CGCGGATCCAATTAGAGCCTGGTCCTCCTTC-3′).

In situ hybridizations on cryosections (30 µm) were performed according to previously described prodedure^68^. In short, hybridizations were carried out at 400 ng ml^−1^ final concentration (cRNA probes) overnight at 64 °C. The digoxigenin-labeled cRNAs were detected by anti-digoxigenin antibody Fab fragments coupled to alkaline phosphatase (Roche) and NBT/BCIP solution (nitroblue tetrazolium/5-bromo-4-chloro-3-indolyl phosphate, Roche). Images were acquired using Leica SCN400 scanners from Leica Microsystems.

### Immunofluorescence

Brains were perfused transcardially with ice-cold PBS followed by 4% PFA in PBS, post-fixed overnight in 4% PFA in PBS at 4 °C, dehydrated in 30% sucrose in PBS, embedded and frozen at −80 °C in O.C.T. compound, and sectioned with Leica CM3050S. Cryosections were subjected to antigen retrieval, pretreated (0.3% Triton X-100 in PBS), incubated with a blocking solution (10% normal donkey serum, 0.1% Triton X-100, and 0.2% gelatin in PBS), followed by incubation with the primary antibodies overnight at 4 °C. Primary antibodies used were as follows: goat anti-SOX2 (Santa Cruz, sc17320, 1:200), mouse anti-Phospho-Vimentin (Ser55, MBL International, D076-3, 1:500), mouse anti-Phospho-Vimentin (Ser82, MBL International, D095-3, 1:500), rabbit anti-Ki67 (Millipore, AB9260, 1:200), mouse anti-ZO1 (Thermo Fisher Scientific, 33-9100, 1:500), mouse anti-β-Catenin (BD Biosciences, 610153, 1:800), mouse anti-N-Cadherin (BD Biosciences, 610920, 1:800), mouse anti-PH3 (Abcam, ab14955, 1:500), mouse anti-Pericentrin (BD Biosciences, 611814, 1:800), rat anti-BrdU (Abcam, ab6326, 1:800), mouse anti-NeuN (Millipore, MAB377, 1:200), chicken anti-GFP (Aves Labs, GFP-1020, 1:500), rabbit anti-RFP (Rockland, 600-401-379, 1:500), mouse anti-Tuj1 (Covance Research Products, MMS-435P, 1:500), mouse anti-GFAP (Cell Signaling Technology, 3670s, 1:300), mouse anti-Nestin (Aves Labs, NES, 1:200), rabbit anti-TTYH1 (Sigma-Aldrich, HPA023617, 1:300), rabbit anti-FAM107A (Sigma-Aldrich, HPA055888, 1:300), rabbit anti-HMGA2 (Abcam, ab207301, 1:200), rabbit anti-Isl1 (Abcam, ab20670, 1:200), and mouse anti-Nr5a1 (R and D Systems, PP-N1665-00, 1:200). Fluorescent-conjugated secondary antibody were incubated for 2 h at RT. Secondary antibodies used were as follows: donkey anti-mouse, anti-rabbit, anti-chicken, anti-rat, or anti-goat Alexa-594-, Alexa-488-, and Alexa-647-conjugated antibodies (1:500, Invitrogen). Cell nuclei were stained using 4,6-diamidino-2-phenylindole (D1306, Invitrogen). Immunofluorescence images were acquired with Olympus laser confocal microscope and analyzed with FV10-ASW viewer (Olympus), ImageJ (NIH), and Photoshop (Adobe).

GFAP-GFP transgenic mice (JAX Stock No: 003257) that expressed GFP under the control of GFAP promoter was used to visualize the topographical features of the RG in the developing hypothalamus and we enhance the GFP fluorescent signal with GFP antibody (chicken anti-GFP Aves Labs, GFP-1020) immunostaining. In brief, brains of embryonic GFAP-GFP transgenic mice at E12.5 or E13.5 were dissected out and post-fixed in 4% PFA overnight at 4 °C followed by 30% sucrose dehydration. Coronal sections (40–50 μm in thickness) were prepared by frozen sectioning and incubated for a blocking solution, followed by incubation with the primary antibodies overnight at 4 °C.

For defining the cell fate after monitoring hmRG cell divisions in real time, we performed immunostaining with cell-type-specific markers. In brief, tissue sample with CMV-GFP-labeled cells were first fixed overnight in 4% PFA. For immunostaining, brain slices were incubated in 0.1% Triton-X PBS with 5% donkey serum (DS) for 10 min, then incubated in PBS (5% DS, 0.1% triton-X) with primary antibodies for at least 36 h at 4 °C. Primary antibodies were goat anti-SOX2 (Santa Cruz, sc17320) and mouse anti-NeuN (Millipore, MAB377). Then rinsed with PBS for 10 min 3 times; after rinsing, brain slices were incubated with secondary antibodies donkey anti-mouse 594 (Invitrogen, A-21203) and donkey anti-goat 647 (Invitrogen, A21447). Fluorescence images were scanned by confocal microscopy (Olympus FV1000).

### Quantification and statistical analysis

Data are presented as mean values ± SEM. The quantification graphs were made using the GraphPad Prism software. Sample size (*n*) for each analysis can be found in the figure legends.

### Reporting summary

Further information on research design is available in the Nature Research Reporting Summary linked to this article.

## Supplementary information

Supplementary Information

Supplementary Movie 1

Supplementary Movie 2

Supplementary Movie 3

Supplementary Movie 4

Supplementary Movie 5

Supplementary Movie 6

Supplementary Movie 7

Supplementary Movie 8

Reporting Summary

## Data Availability

The accession number for the RNA sequencing data reported in this paper is GSE118487. We performed the analysis of gene co-expression networks by STRING database (https://string-db.org/). Source data are provided with this paper.

## Source data

Source Data
