# Supplementary material for: Cellular and molecular properties of neural progenitors in the developing mammalian hypothalamus
Source: Nat Commun. 2020 Aug 13;11:4063. doi: 10.1038/s41467-020-17890-2 (PMC7426815; doi:10.1038/s41467-020-17890-2)
Supplement: Supplementary file 1 — Supplementary Information [file 41467_2020_17890_MOESM1_ESM.pdf]

Supplemental Information

Cellular and molecular properties of neural progenitors in the developing mammalian hypothalamus

Zhou et al.

Supplementary figures 1-6

Supplementary Figure 1

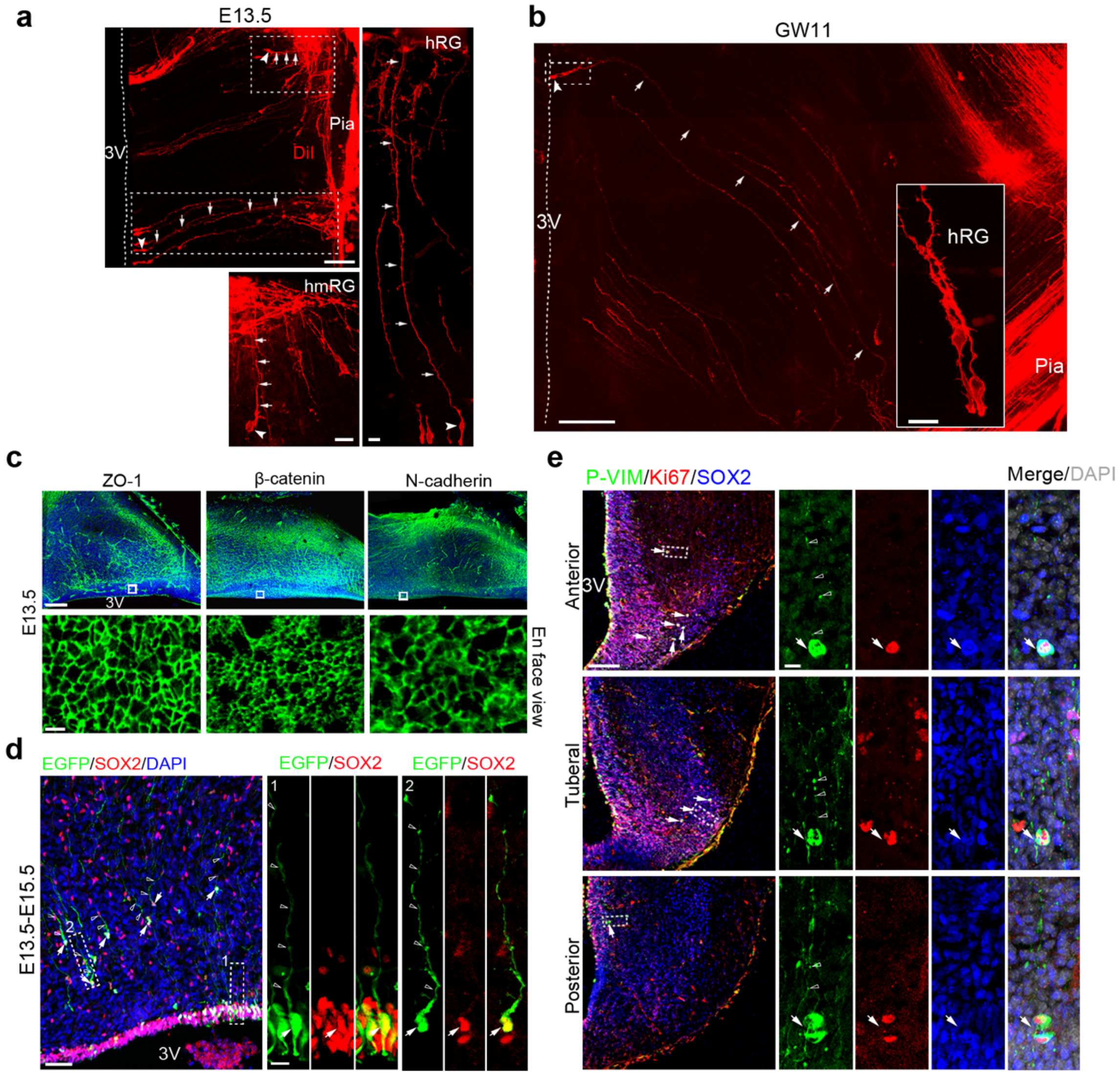

Supplementary Figure 1. Characterization of hypothalamic progenitor

(**a,b**) Labeling hRG cells and hmRG cells by applying DiI on the pia surface of the developing hypothalamus at E13.5 mouse (a, *n* = 3 independent experiments) and GW11 human (b, *n* = 3 independent experiments). The hRG cells in the VZ show various morphology. Arrowheads indicate cell body. Filled arrows indicate basal fibers. Dashed lines pinpoint the surface of the third ventricle. 3V, the third ventricle. Scale bars, 100 μm

(top left), 20 μm (bottom left and right) in **a**; 200 μm (left), 20 μm (right) in **b**.

(**c**) Representative image of adhesion molecule (ZO-1, β-catenin and N-cadherin) at the apical ventricular endfeet of hRG cells, exhibiting ring-like structure from an 'en face' view of the third ventricular surface at E13.5 (*n* = 3 independent experiments). DAPI, blue. Scale bars, 100 μm (top), 5μm (bottom).

(**d**) Representative images of hRG (box 1) and hmRG cells (box 2) 2 d after electroporation of EGFP-expressing vector in utero and staining with radial glial progenitor marker SOX2 (red) (*n* = 3 independent experiments). DNA, blue. Arrows indicate double-positive hRG and hmRG cells. Open arrowheads indicate basal processes. Scale bars, 50 μm (left), 10 μm (right).

(**e**) Phosphovimentin (green) labels hmRG cells in mitosis throughout the rostral-caudal developing hypothalamus including the anterior, tuberal and posterior region (*n* = 3 independent experiments). Higher magnification images of the outlined hmRG cells with basal fibers are shown in right. The hmRG cells (P-VIM, green) co-stained with radial glial progenitor marker SOX2 (blue) and proliferative marker Ki67 (red). DAPI, gray. Filled arrows point to triple positive hmRG cells. Open arrowheads indicate basal processes. Scale bars, 100 μm (left), 10 μm (right).

Supplementary Figure 2

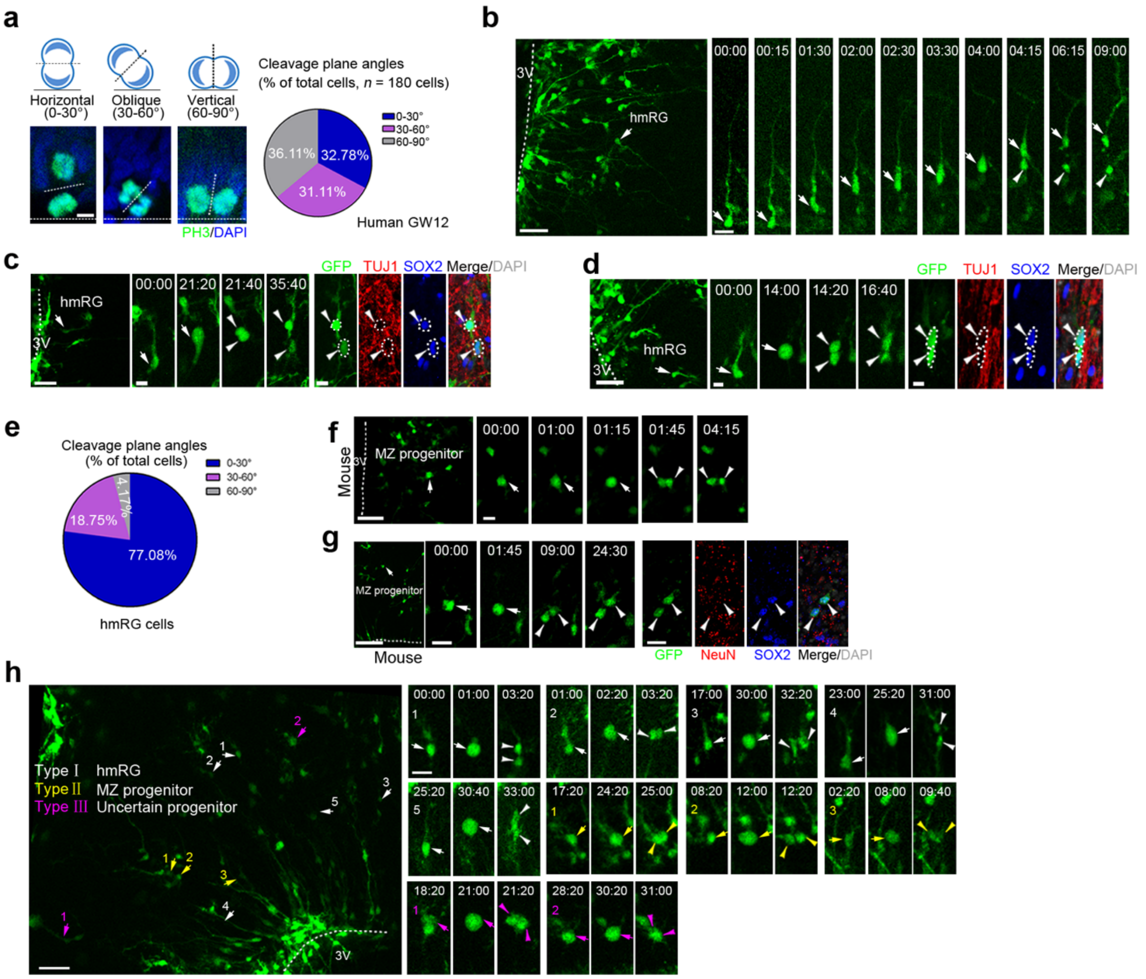

Supplementary Figure 2. Progenitor divisions in the developing hypothalamus

(**a**) Measurement of the percentage of cleavage plane orientation as the angle between the cleavage plane and the 3V surface by immunostaining PH3 at GW12, grouped into vertical (60-90°), oblique (30-60°) and horizontal (0-30°). *n* = 180 cells. Scale bar, 5 μm.

(**b**) The representive hmRG undergoes mitotic somal translocation directly before cell division in mice (*n* = 7 independent experiments). Arrows indicate monopolar RG cells and arrowheads indicate a non-hmRG daughter. Time stamp, h:min. Scale bars, 50 μm (left) and 20 μm (right).

(**c**) Asymmetric hmRG cell division produced progenitor-like daughter cells (SOX2+, but TUJ1-) and neuronal daughter cells (TUJ1+) (*n* = 3 independent experiments). Time stamp, h:min. Scale bars, 50 μm (left), 10 μm (middle and right).

(**d**) A hmRG cell division gave rise to two progenitor-like daughter cells (SOX2+, but TUJ1-, *n* = 4 independent experiments). Time stamp, h:min. Scale bars, 50 μm (left), 10 μm (middle and right).

(**e**) Quantification of the cleavage plane angles in mice hmRG (*n* = 48) after time-lapse imaging. Source data are supplied as a Source data file.

(**f**) GFP-labeled MZ progenitor cells in mice hypothalamus divide in place without nuclear translocation (*n* = 10 independent experiments). White arrows indicate the MZ progenitors. White arrowheads indicate the daughter cells. Scale bars, 50 μm (left) and 20 μm (right).

(**g**) A MZ progenitor cell (arrows) divided directly without nucleus translocation. Both of the MZ progenitor daughter cells (arrowheads, *n* = 3 independent experiments) were $SOX2^{+}$, but $NeuN^{-}$. DNA, gray. Time stamp, h: min. Scale bars, 100 μm (left), 20 μm (middle and right).

(**h**) Multiple progenitor cells located in MZ actively divided (*n* = 6 independent experiments). There are three types of progenitor behaviors, including classic MST of hmRG cells (Type I, white arrows), in situ division of MZ progenitor cells (Type II, yellow arrows) and uncertain behavior of MZ progenitors (Type III, magenta arrows). Arrows indicate progenitors and arrowheads indicate daughter cells. Time stamp, h:min. Scale bars, 50 μm (left), 20 μm (right). Source data are supplied as a Source data file.

Supplementary Figure 3

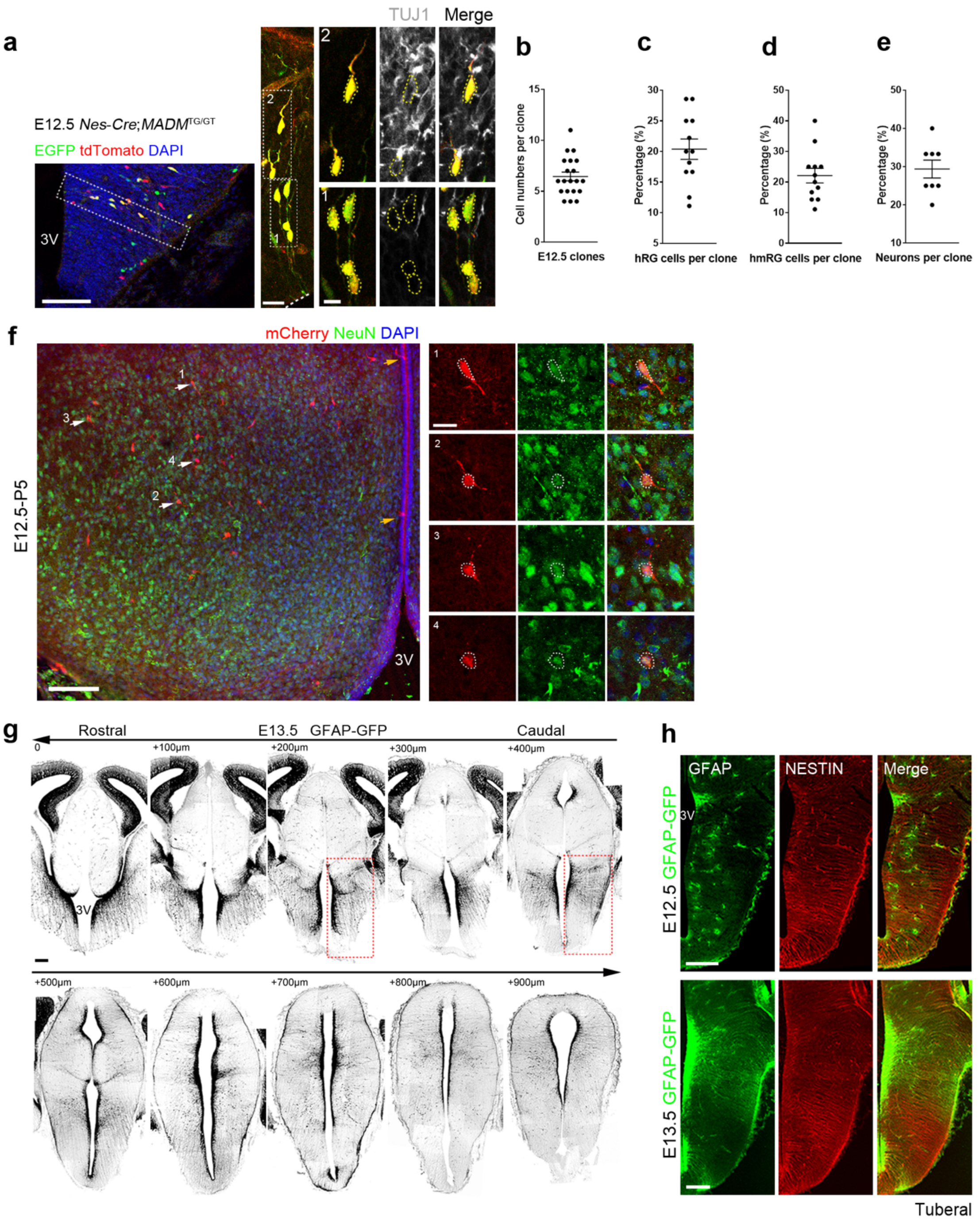

Supplementary Figure 3. Radial clusters in early embryonic hypothalamus neurogenesis

(**a**) Cellular composition of MADM-labeled embryonic hypothalamic clones (n = 3

independent experiments). Radial cluster labeled by MADM stained TUJ1 (gray). Yellow dotted lines indicate cell bodies. Scale bars, 100 µm (left), 20 µm (middle and right).

(**b**) Quantification of MADM clone size at E12.5 (*n* = 20 clones).

(**c-e**) Percentage of hRG cells (*n* =12 clones, c), hmRG cells (*n* = 12 clones, d) and neurons (*n* = 8 clones, e) in MADM-labeled hypothalamic clones respectively.

(**f**) Images of P5 mCherry-positive hypothalamic progeny cells (red) labeled at E12.5 and stained with antibody against NeuN (green, neuronal marker) (*n* = 3 independent experiments). Yellow arrow indicates radial glia cells located at the third ventricle. White arrow indicates the daughter neuronal cells sparsely distributed. High-magnification images of labeled NeuN positive neurons were shown in the right. Scale bars, 100 µm (left), 20 µm (right, areas 1-4). 3V, the third ventricle.

(**g**) Representative rostral-to-caudal serial coronal sections of GFAP expression at E13.5 GFAP-GFP mice, varying dorso-ventral GFAP level in the ventricular zone of the third ventricle. Outlined images are shown in Fig. 3d. Scale bar, 200 µm.

(**h**) Staining of hypothalamic tissue sections for NESTIN (red) and GFP (GFAP, green) at E12.5 and E13.5 GFAP-GFP mice (*n* = 3 independent experiments). Scale bars, 200 µm (top and bottom). Data are presented as mean values ± SEM. Source data are supplied as a Source data file.

Supplementary Figure 4

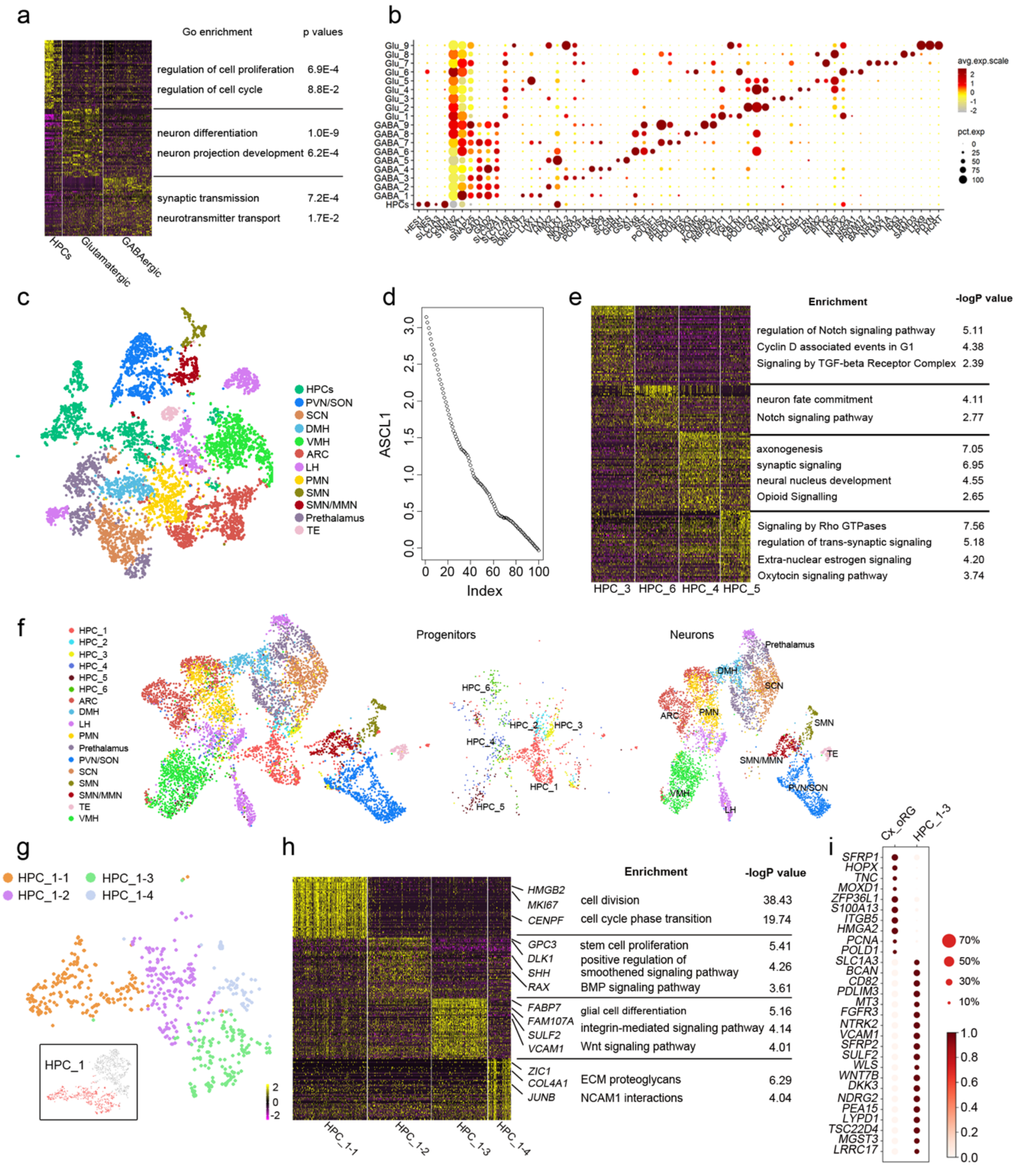

Supplementary Figure 4. Cell subtypes in developing human hypothalamus

(**a**) Heat map shows blocks of genes enriched in each major cell type. Enriched gene ontology terms were displayed in right. Benjamini correction for Go analysis in

DAVID7.0

(**b**) Differential gene expression by hypothalamic progenitors, glutamatergic neurons (Glu 1-9) and GABAergic neurons (GABA 1-9).

(**c**) tSNE plot of developing hypothalamic and adjacent regions in human.

(**d**) Expression of marker gene as a function of the position along the maturation trajectory.

(**e**) Heat map of differentially expressed genes in the progenitor subtypes with different maturation stage. The key enriched gene ontology terms were represented in the right. The enrichment analyses were performed using Metascape with p value cutoff <0.01.

(**f**) Integration of progenitor and neurons was visualized by UMAP. The right panels show the separated UMAP of progenitor and neuronal cells, respectively.

(**g**) Representation of subtypes of radial glia cell (HPC_1) in developing hypothalamus using t-SNE.

(**h**) Heat map of differentially expressed genes of subclasses in HPC_1. Different expression genes in each type were shown on the right of each heat map panel with enriched gene ontology terms. The enrichment analyses were performed using Metascape with p value cutoff <0.01.

(**i**) Dot plot showing the different gene expression pattern between hypothalamic progenitor (HPC_1-3) and cortical oRG cells. Source data are supplied as a Source data file.

Supplementary Figure 5

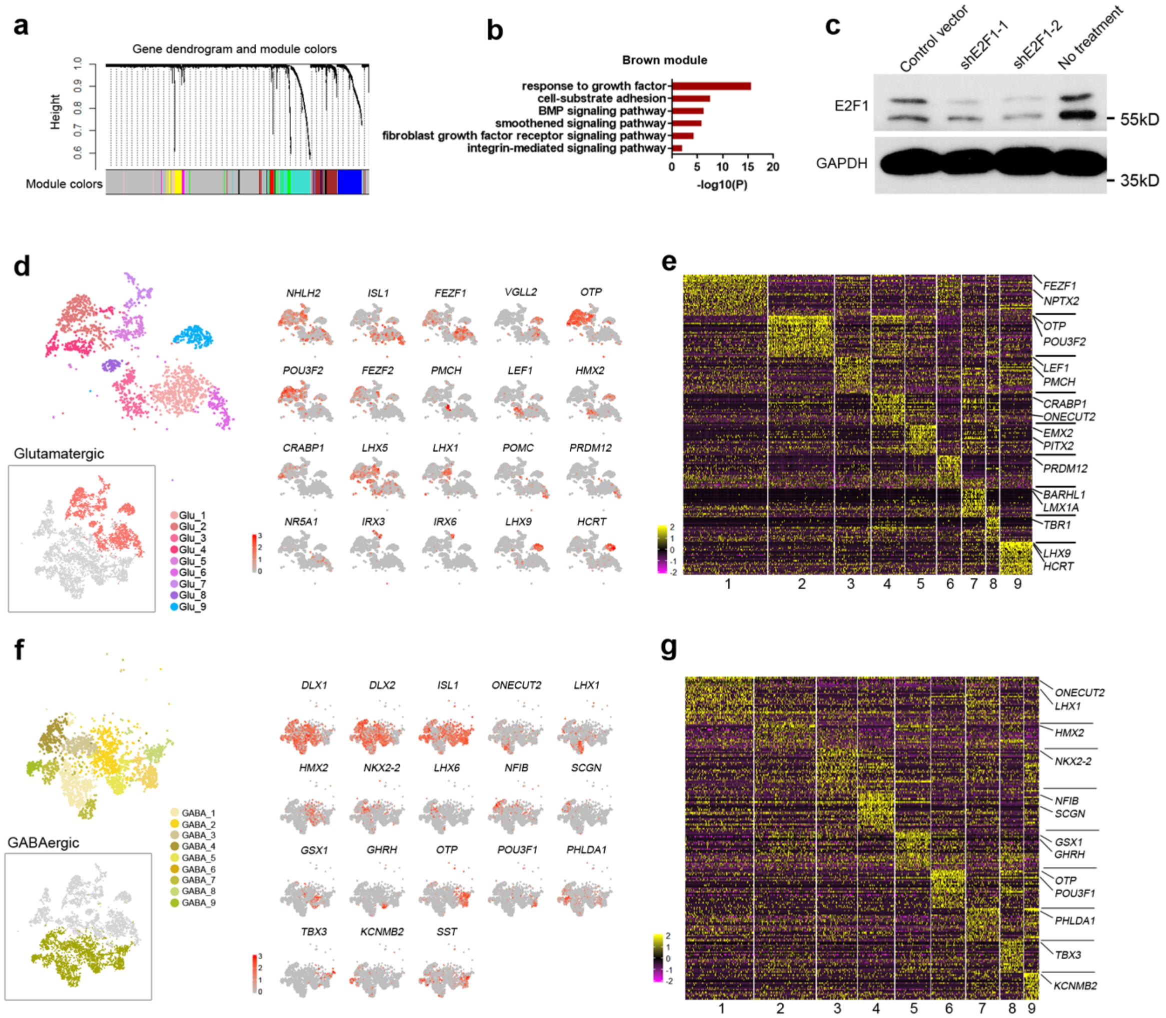

Supplementary Figure 5 Molecular diversity of subgroups of hypothalamic progenitor cells

(**a**) Gene clustering and module identification. Each co-expression cluster has a different color.

(**b**) Major gene ontology terms or pathways associated with the brown modules. The enrichment analyses were performed by Metascape with *p* value cutoff <0.01.

(**c**) Detection of E2F1 (60 kD) protein levels in HEK293T cells transfected with

shE2F1-1, shE2F1-2 plasmids, with a scramble sequence as the control. GAPDH (38 kD) was detected as a loading control (n = 3 independent experiments).

(**d**) tSNE plot showing the 9 glutamatergic neuron subtypes identified in hypothalamus. Expression patterns of marker genes for different subtypes displayed on t-SNE plots (grey, no expression; red, increased relative expression).

(**e**) Heat map of differentially expressed genes of subtypes of Glutamatergic neurons. Marker genes for each subtype are shown on the right of each heatmap panel. Purple to yellow indicates a gradient from low to high gene expression.

(**f**) tSNE plot showing the 9 GABAergic neuron subtypes. Expression patterns of marker genes for different subtypes displayed on t-SNE plots (grey, no expression; red, increased relative expression).

(**g**) Heat map of differentially expressed genes of subtypes of GABAergic neurons. Marker genes for each subtype are shown. Purple to yellow indicates a gradient from low to high gene expression. Source data are supplied as a Source data file.

Supplementary Figure 6

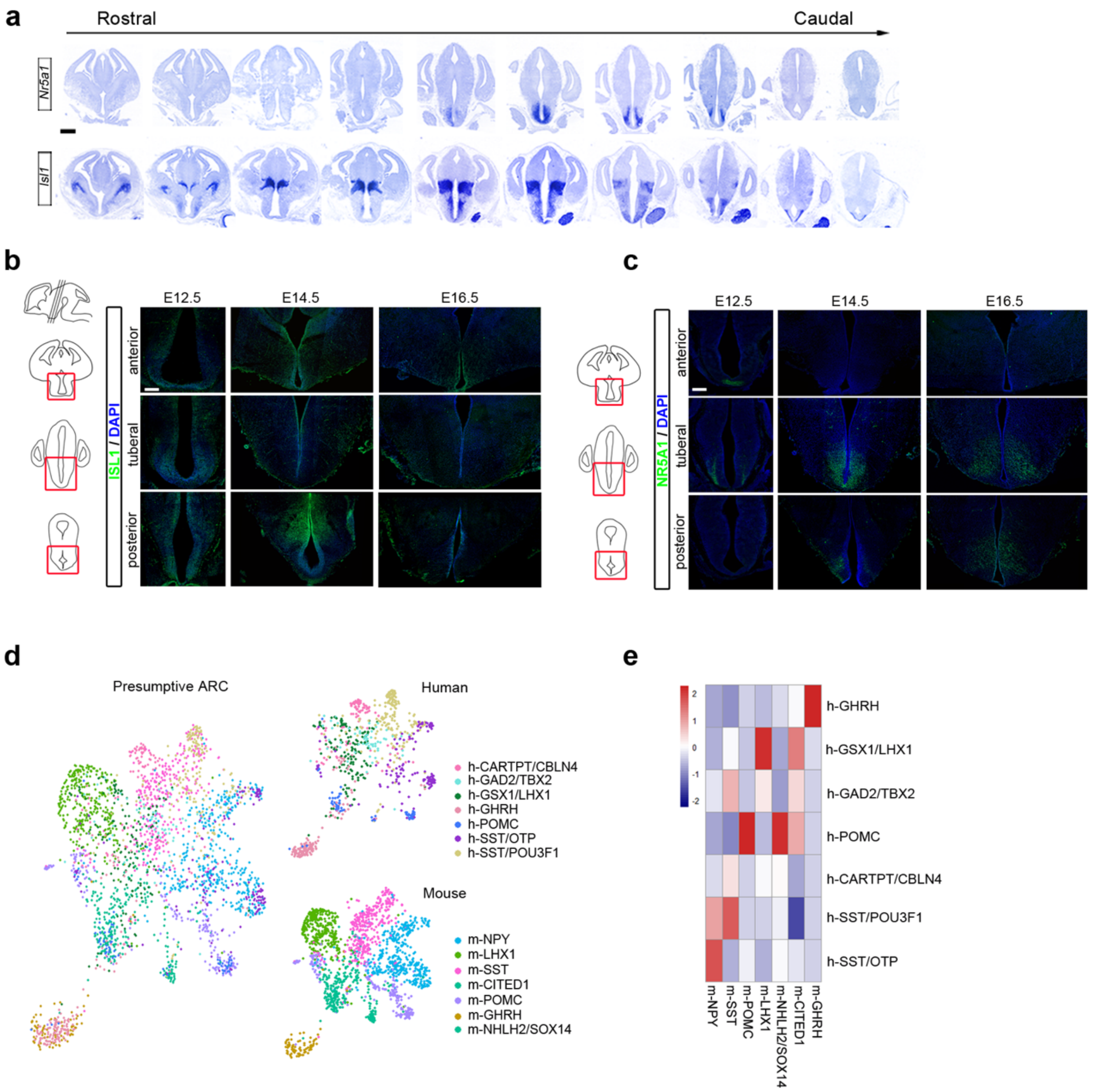

Supplementary Figure 6 Zone-specific transcription factors in the developing hypothalamus

(**a**) In situ hybridization with marker genes including *Nr5a1* and *Isl1* in rostral-to-caudal serial coronal sections of hypothalamus at E12.5. Scale bar, 500 μm.

(**b,c**) Immunostaining of marker genes in developing mice hypothalamus. Schematic diagram illustrating that expression pattern was viewed in anterior, tuberal and posterior

domain in the mouse developing hypothalamus (E12.5, E14.5 and E16.5, *n* = 3 independent experiments). ISL1 (b) and NR5A1 (c) were differently spatially expressed in MZ along rostral-to-caudal axis and clearer compartmentation showed up as the hypothalamus developed. DAPI, blue. Scale bar, 200 μm in (b, c).

(**d**) After being integrated, the neuronal subset distributions in presumptive ARC between human and mouse were visualized in UMAP. Each dot represents a single cell and is colored according to the cell types.

(**e**) The transcriptomic correlations between the ARC neuronal subclusters between human and mouse were visualized via heatmap.
