# Supplementary material for: Cellular and molecular properties of neural progenitors in the developing mammalian hypothalamus
Source: Nat Commun. 2020 Aug 13;11:4063. doi: 10.1038/s41467-020-17890-2 (PMC7426815; doi:10.1038/s41467-020-17890-2)
Supplement: Supplementary file 10 — Reporting Summary [file 41467_2020_17890_MOESM10_ESM.pdf]

nature research

Corresponding author(s): Xiaoqun Wang

Last updated by author(s): 7/19/2020

# Reporting Summary

Nature Research wishes to improve the reproducibility of the work that we publish. This form provides structure for consistency and transparency in reporting. For further information on Nature Research policies, see our Editorial Policies and the Editorial Policy Checklist.

## Statistics

For all statistical analyses, confirm that the following items are present in the figure legend, table legend, main text, or Methods section.

| n/a | Confirmed | |
|---|---|---|
| ☐ | ☒ | The exact sample size ($n$) for each experimental group/condition, given as a discrete number and unit of measurement |
| ☐ | ☒ | A statement on whether measurements were taken from distinct samples or whether the same sample was measured repeatedly |
| ☐ | ☒ | The statistical test(s) used AND whether they are one- or two-sided<br>*Only common tests should be described solely by name; describe more complex techniques in the Methods section.* |
| ☒ | ☐ | A description of all covariates tested |
| ☐ | ☒ | A description of any assumptions or corrections, such as tests of normality and adjustment for multiple comparisons |
| ☐ | ☒ | A full description of the statistical parameters including central tendency (e.g. means) or other basic estimates (e.g. regression coefficient) AND variation (e.g. standard deviation) or associated estimates of uncertainty (e.g. confidence intervals) |
| ☐ | ☒ | For null hypothesis testing, the test statistic (e.g. $F$, $t$, $r$) with confidence intervals, effect sizes, degrees of freedom and $P$ value noted<br>*Give P values as exact values whenever suitable.* |
| ☒ | ☐ | For Bayesian analysis, information on the choice of priors and Markov chain Monte Carlo settings |
| ☒ | ☐ | For hierarchical and complex designs, identification of the appropriate level for tests and full reporting of outcomes |
| ☐ | ☒ | Estimates of effect sizes (e.g. Cohen's $d$, Pearson's $r$), indicating how they were calculated |

*Our web collection on statistics for biologists contains articles on many of the points above.*

## Software and code

Policy information about availability of computer code

| | |
|---|---|
| Data collection | Image data were collected using FV10-ASW software version 4.1 on Fluoview FV1000 (Olympus); Live-imaging data were acquired using inverted laser scanning microscope Fluoview FV1000 (Olympus); RNA samples were sequenced on Illumina platform for sequencing with 150bp pair-end reads. |
| Data analysis | R software version 3.5.1; Cellranger v2.0.1; Existing R package: Seurat v2.3.4, WGCNA v1.66, pheatmap v 1.0.12, princurve v 2.1.4, diffusion Map v1.2.0, Matrix v1.2-14, dplyr, 0.8.4, ggplot2 v3.2.0; David 6.7, GraphPad Prism 6.0, ImageJ software 1.49v; See Methods for details on how each software is used. |

For manuscripts utilizing custom algorithms or software that are central to the research but not yet described in published literature, software must be made available to editors and reviewers. We strongly encourage code deposition in a community repository (e.g. GitHub). See the Nature Research guidelines for submitting code & software for further information.

## Data

Policy information about availability of data

All manuscripts must include a data availability statement. This statement should provide the following information, where applicable:

- Accession codes, unique identifiers, or web links for publicly available datasets
- A list of figures that have associated raw data
- A description of any restrictions on data availability

The scRNA-seq data used in this study have been deposited in the Gene Expression Omnibus (GEO) under accession numbers GSE118487. We perform the analysis of gene co-expression networks by STRING database (https://string-db.org/).



April 2020

# Field-specific reporting

Please select the one below that is the best fit for your research. If you are not sure, read the appropriate sections before making your selection.

☒ Life sciences ☐ Behavioural & social sciences ☐ Ecological, evolutionary & environmental sciences

For a reference copy of the document with all sections, see nature.com/documents/nr-reporting-summary-flat.pdf

# Life sciences study design

All studies must disclose on these points even when the disclosure is negative.

| | |
|---|---|
| Sample size | A sample size of each measurement was used by the practical limitations of the protocol utilized and accepted according to previously published criteria for in vitro or in vivo biological experiments. For quantitative experiments, while assuring reproducibility and accuracy of quantitative result, we did 3-10 individual experiments. See Figure Legends for details on exact sample size for each result. For scRNA-seq, the sample size was determined by availability of human tissues. Final dataset scale was determined according to the quality control criteria as described in the methods. |
| Data exclusions | As described in the paper, Cells detected with less than 800 genes and were removed as low quality cells. Genes which only expressed in fewer than 7 cells (0.1% of total cell number) were excluded as recommended by Seurat (Ver 2.3.4 ). Cells that expressed haemoglobin genes (HBA1,HBA2, HBB, HBD, HBE1, HBG1, HBG2, HBM, HBQ1 and HBZ) were also removed . In short, low quality cells with less genes and blood cells were excluded from further analysis. |
| Replication | All replications were successful by at least three independent experiment. |
| Randomization | For animal experiments, the samples were allocated into each experimental groups based on the genotype and age. No sex selection. For human samples, there were no sex selection. |
| Blinding | Investigators were blind to group allocation during data collection and analysis. |

# Reporting for specific materials, systems and methods

We require information from authors about some types of materials, experimental systems and methods used in many studies. Here, indicate whether each material, system or method listed is relevant to your study. If you are not sure if a list item applies to your research, read the appropriate section before selecting a response.

## Materials & experimental systems

| n/a | Involved in the study | |
|---|---|---|
| ☐ | ☒ | Antibodies |
| ☐ | ☒ | Eukaryotic cell lines |
| ☒ | ☐ | Palaeontology and archaeology |
| ☐ | ☒ | Animals and other organisms |
| ☐ | ☒ | Human research participants |
| ☒ | ☐ | Clinical data |
| ☒ | ☐ | Dual use research of concern |

## Methods

| n/a | Involved in the study | |
|---|---|---|
| ☒ | ☐ | ChIP-seq |
| ☒ | ☐ | Flow cytometry |
| ☒ | ☐ | MRI-based neuroimaging |

## Antibodies

| | |
|---|---|
| Antibodies used | For immunostaining, the following antibodies to the following proteins were used:<br>Primary:<br>Goat polyclonal to SOX2, Santa Cruz, Catalog # sc17320,H1406,1:200<br>Mouse monoclonal to Phospho-Vimentin (Ser 55), MBL International, Catalog # D076-3, 033, 1:500<br>Mouse monoclonal anti- Phospho-Vimentin (Ser 82), MBL International, Catalog # D095-3, 020, 1:500<br>Rabbit polyclonal to Ki67, Millipore, Catalog # AB9260, 1:200<br>Mouse monoclonal [ZO1-1A12] to ZO1, Thermo Fisher Scientific, Catalog # 33-9100,1:500<br>Mouse monoclonal [14/Beta-Catenin] to β-Catenin, BD Biosciences, Catalog # 610153, G22121,1:800<br>Mouse monoclona [32/N-Cadherin] to N-Cadherin, BD Biosciences, Catalog # 610920, 2307890,1:800<br>Mouse monoclonal [mAbcam 14955] to PH3, Abcam, Catalog # ab14955, 1:500<br>Mouse monoclonal [30/Pericentrin] to Pericentrin, BD Biosciences, Catalog # 611814,1:800<br>Rat monoclonal [BU1/75 (ICR1)] to BrdU, Abcam, Catalog # ab6326,1:800<br>Mouse monoclonal [A60] to NeuN, Millipore, Catalog # MAB377,2654334, 1:200<br>Chicken polyclonal to GFP, AvesLabs, Catalog # GFP-1020,1:500<br>Rabbit polyclonal to RFP, Rockland, Catalog # 600-401-379,1:500<br>Mouse monoclonal to Tuj1, Covance Research Products, Catalog # MMS-435P, E10359JF, 1:500<br>Mouse monoclonal [GA5] to GFAP, Cell Signaling Technology, Catalog # 3670s, 6, 1:300 |

Mouse to Nestin, Aves Labs, Catalog # NES, 1:200
Rabbit polyclonal to TTYH1, Sigma-Aldrich, Catalog # HPA023617, R10755, 1:300
Rabbit polyclonal to FAM107A, Sigma-Aldrich, Catalog # HPA055888, B115825, 1:300
Rabbit monoclonal [EPR18114] to HMGA2,Abcam, Catalog # ab207301, 1:200
Rabbit polyclonal to Isl1, Abcam, Catalog # ab20670,1:200
Mouse monoclonal to Nr5a1, R and D Systems, Catalog # PP-N1665-00, 1:200
Rabbit polyclonal to E2F1, Santa Cruz, Catalog # sc-193, H2818, 1:1000
HRP-labeled anti-GAPDH monoclonal antibody, KangChen Bio-tech, KC-5G5, 1:2000

Secondary:
Alexa Fluor 488 Conjuated Affinipure Donkey anti-Rat IgG(H+L), Invitrogen, A-21208, 1:500
Alexa Fluor 488 Conjuated Affinipure Donkey anti-Mouse IgG(H+L) , Invitrogen, A-21202, 1:500
Alexa Fluor 488 Conjuated Affinipure Donkey anti-Rabbit IgG(H+L), Invitrogen, A-21206, 1:500
FITC Conjuated Affinipure Donkey anti-Chicken IgY(H+L),Abcam, ab63507, 1:800
Alexa Fluor 594 Conjuated Affinipure Donkey anti-Rabbit IgG(H+L), Invitrogen, A21207, 1:500
Alexa Fluor 594 Conjuated Affinipure Donkey anti-MouseI gG(H+L), Invitrogen, A-21203, 1:500
Alexa Fluor 594 ConjuatedAffinipure Donkey anti-Goat IgG(H+L), Invitrogen, A-11058, 1:500
Alexa Fluor 647 Conjuated Affinipure Donkey anti-Goat IgG(H+L), Invitrogen, A21447, 1:500
Alexa Fluor 647 Conjuated Affinipure Donkey anti-Rabbit IgG(H+L), Invitrogen, A-31573, 1:500
Alexa Fluor 647 Conjuated Affinipure Donkey anti-Mouse IgG(H+L), Invitrogen, A-31571, 1:500

Goat anti-Rabbit HRP, Abcam, ab136817, 1:1000

Validation

All antibodies were obtained from commercial vendors. All antibodies and their applications used in this study have been validated in previously published studies as listed in their manufactures' websites.
Primary:
Goat polyclonal to SOX2, Santa Cruz, Catalog # sc17320
Species Reactivity: Bovine, Chicken, Human, Mouse, Pig, Rat, Worm ;
Applications: ELISA, IF, IHC, IP, WB
Ding, W., Wu, Q., Sun, L., Pan, N.C., and Wang, X. (2019). Cenpj regulates cilia disassembly and neurogenesis in the developing mouse cortex. J Neurosci.

Mouse monoclonal to Phospho-Vimentin (Ser 55), MBL International, Catalog # D076-3
Species Reactivity: Human, Mouse, Rat
Applications: ELISA, IH, IC, WB
Miyata T et al. Asymmetric production of surface-dividing and non-surface-dividing cortical progenitor cells. Development. 131, 3133-45 (2004)

Mouse monoclonal anti- Phospho-Vimentin (Ser 82), MBL International, Catalog # D095-3
Species Reactivity: Human, Mouse, Rat
Applications: IC, WB
Liu, J., Liu, W., Yang, L., Wu, Q., Zhang, H., Fang, A., Li, L., Xu, X., Sun, L., Zhang, J., et al. (2017). The Primate-Specific Gene TMEM14B Marks Outer Radial Glia Cells and Promotes Cortical Expansion and Folding. Cell Stem Cell 21, 635-649 e638.

Rabbit polyclonal to Ki67, Millipore, Catalog # AB9260
Species Reactivity: Human, Mouse, Rat
Applications: IH(P), WB
Liu, J., Liu, W., Yang, L., Wu, Q., Zhang, H., Fang, A., Li, L., Xu, X., Sun, L., Zhang, J., et al. (2017). The Primate-Specific Gene TMEM14B Marks Outer Radial Glia Cells and Promotes Cortical Expansion and Folding. Cell Stem Cell 21, 635-649 e638.

Mouse monoclonal [ZO1-1A12] to ZO1, Thermo Fisher Scientific, Catalog # 33-9100
Species Reactivity: Bovine, Chicken, Dog, Fish, Guinea pig, Hamster, Human, Mammal, Mouse, Non-human primate, Pig, Rabbit, Rat, Reptile, Rhesus monkey, Sheep, Tag, Virus, Xenopus, Zebrafish
Applications: ELISA, ICC, IF, WB
Revinski, D.R., and Zaragosi, L.E. (2018). CDC20B is required for deuterosome-mediated centriole production in multiciliated cells. 9, 4668.

Mouse monoclonal [14/Beta-Catenin] to β-Catenin, BD Biosciences, Catalog # 610153
Species Reactivity: Human (QC Testing) Mouse, Rat, Dog, Chicken (Tested in Development)
Applications: Western blot (Routinely Tested) .Immunohistochemistry, Immunoprecipitation, Immunofluorescence (Tested During Development)
Eger, A., Stockinger, A., Schaffhauser, B., Beug, H., and Foisner, R. (2000). Epithelial mesenchymal transition by c-Fos estrogen receptor activation involves nuclear translocation of beta-catenin and upregulation of beta-catenin/lymphoid enhancer binding factor-1 transcriptional activity. J Cell Biol 148, 173-188.

Mouse monoclona [32/N-Cadherin] to N-Cadherin, BD Biosciences, Catalog # 610920
Species Reactivity: Human (QC Testing) Mouse, Rat, Chicken (Tested in Development)
Applications: Western blot (Routinely Tested) Immunofluorescence (Tested During Development) Immunoprecipitation (Reported)
Nürnberger, J., Bacallao, R.L., and Phillips, C.L. (2002). Inversin forms a complex with catenins and N-cadherin in polarized epithelial cells. Mol Biol Cell 13, 3096-3106.

Mouse monoclonal [mAbcam 14955] to PH3, Abcam, Catalog # ab14955
Species Reactivity: Mouse, Rat, Human, Xenopus laevis, Arabidopsis thaliana, Drosophila melanogaster, Indian muntjac, African green monkey, Oncopeltus
Applications: IHC-P, ELISA, ICC/IF, IP, IHC-Fr, Flow Cyt, WB
Ehrmann, I., Crichton, J.H., Gazzara, M.R., James, K., Liu, Y., Grellscheid, S.N., and Curk, T. (2019). An ancient germ cell-specific RNA-

binding protein protects the germline from cryptic splice site poisoning. 8.

Mouse monoclonal [30/Pericentrin] to Pericentrin, BD Biosciences, Catalog # 611814
Species Reactivity: Mouse (QC Testing)
Applications: Western blot (Routinely Tested) Immunofluorescence (Tested During Development)
Young, A., Dictenberg, J.B., Purohit, A., Tuft, R., and Doxsey, S.J. (2000). Cytoplasmic dynein-mediated assembly of pericentrin and gamma tubulin onto centrosomes. Mol Biol Cell 11, 2047-2056.

Rat monoclonal [BU1/75 (ICR1)] to BrdU, Abcam, Catalog # ab6326
Applications: ICC/IF, IHC-FoFr, IHC-P, IHC-Fr, Flow Cyt, IHC-FrFl
Ding, W., Wu, Q., Sun, L., Pan, N.C., and Wang, X. (2019). Cenpj regulates cilia disassembly and neurogenesis in the developing mouse cortex. J Neurosci.

Mouse monoclonal [A60] to NeuN, Millipore, Catalog # MAB377
Species Reactivity: Av, Ch, Ft, H, M, Po, R, Sal
Applications: FC, IC, IF, IH, IH(P), IP and WB.
Lipinski, M., Muñoz-Viana, R., del Blanco, B., Marquez-Galera, A., Medrano-Relinque, J., Caramés, J.M., Szczepankiewicz, A.A., Fernandez-Albert, J., Navarrón, C.M., Olivares, R., et al. (2020). KAT3-dependent acetylation of cell type-specific genes maintains neuronal identity in the adult mouse brain. Nature Communications 11, 2588.

Chicken polyclonal to GFP, AvesLabs, Catalog # GFP-1020
Applications: ELISA, ICC, IHC, WB
Pan, N.C., Fang, A., Shen, C., Sun, L., Wu, Q., and Wang, X. (2018). Early Excitatory Activity-Dependent Maturation of Somatostatin Interneurons in Cortical Layer 2/3 of Mice. Cereb Cortex.

Rabbit polyclonal to RFP, Rockland, Catalog # 600-401-379
Applications: ELISA, Flow Cytometry, Western Blot, Immunohistochemistry,IF Microscopy
Fu, D.J., Wang, L., Chouairi, F.K., Rose, I.M., Abetov, D.A., and Miller, A.D. (2020). Gastric squamous-columnar junction contains a large pool of cancer-prone immature osteopontin responsive Lgr5(-)CD44(+) cells. 11, 84.

Mouse monoclonal to Tuj1, Covance Research Products, Catalog # MMS-435P
Species Reactivity: Human, Mouse, Rat
Applications:IHC-P - Quality tested, WB, ICC - Validated, FC - Reported in the literature
Wang, X., Tsai, J.W., LaMonica, B., and Kriegstein, A.R. (2011). A new subtype of progenitor cell in the mouse embryonic neocortex. Nat Neurosci 14, 555-561.

Mouse monoclonal [GA5] to GFAP, Cell Signaling Technology, Catalog # 3670s
Species Reactivity: Human, Mouse, Rat
Applications:Western Blotting, Immunohistochemistry (Paraffin), Immunofluorescence (Frozen), Immunofluorescence (Immunocytochemistry), Flow Cytometry
Schlüter, A., Aksan, B., Diem, R., Fairless, R., and Mauceri, D. (2019). VEGFD Protects Retinal Ganglion Cells and, consequently, Capillaries against Excitotoxic Injury. Molecular therapy Methods & clinical development 17, 281-299.

Mouse to Nestin, Aves Labs, Catalog # NES
Species Reactivity: Mouse, Rat
Applications: ICC, IHC, WB
Nakamuta, S., Yang, Y.T., and Wang, C.L. (2017). Dual role for DOCK7 in tangential migration of interneuron precursors in the postnatal forebrain. 216, 4313-4330.

Rabbit polyclonal to TTYH1, Sigma-Aldrich, Catalog # HPA023617
Species Reactivity: human
Applications:Immunohistochemistry
Jung, E., Osswald, M., Blaes, J., Wiestler, B., Sahm, F., Schmenger, T., Solecki, G., Deumelandt, K., Kurz, F.T., Xie, R., et al. (2017). Tweety-Homolog 1 Drives Brain Colonization of Gliomas. The Journal of neuroscience : the official journal of the Society for Neuroscience 37, 6837-6850.

Rabbit polyclonal to FAM107A, Sigma-Aldrich, Catalog # HPA055888
Species Reactivity: human
Applications:immunofluorescence, immunohistochemistry
Pollen, A.A., Nowakowski, T.J., Chen, J., Retallack, H., Sandoval-Espinosa, C., Nicholas, C.R., Shuga, J., Liu, S.J., Oldham, M.C., Diaz, A., et al. (2015). Molecular identity of human outer radial glia during cortical development. Cell 163, 55-67.

Rabbit monoclonal [EPR18114] to HMGA2,Abcam, Catalog # ab207301
Species Reactivity: Human
Applications: WB, IHC-P, ICC/IF
Zhong, S., Zhang, S., Fan, X., Wu, Q., Yan, L., Dong, J., Zhang, H., Li, L., Sun, L., Pan, N., et al. (2018). A single-cell RNA-seq survey of the developmental landscape of the human prefrontal cortex. Nature 555, 524-528.

Rabbit polyclonal to Isl1, Abcam, Catalog # ab20670
Species Reactivity: Mouse, Rat, Human, Apteronotus leptorhynchus
Applications:IHC-FoFr, IHC-P, ICC/IF, IHC-Fr
Qin, J., Wang, M., Zhao, T., Xiao, X., Li, X., Yang, J., Yi, L., Goffinet, A.M., Qu, Y., and Zhou, L. (2020). Early Forebrain Neurons and Scaffold Fibers in Human Embryos. Cereb Cortex 30, 913-928.

Mouse monoclonal to Nr5a1, R and D Systems, Catalog # PP-N1665-00
Species Reactivity: Human

Applications:Western Blot, Immunohistochemistry, Immunoprecipitation
Corman, T.S., Bergendahl, S.E., and Epstein, D.J. (2018). Distinct temporal requirements for Sonic hedgehog signaling in development of the tuberal hypothalamus. In Development (Cambridge, England).

Rabbit polyclonal to E2F1, Santa Cruz, Catalog # sc-193
Species Reactivity: Bos taurus (Bovine)Canis lupus familiaris (Domestic dog)Equus caballus (Horse)Homo sapiens (Human)Mus musculus (House mouse)Rattus norvegicus (Rat)Sus scrofa domesticus (Pig)
Applications:western blot, immunohistochemistry, immunocytochemistry
Morris, E.J., Ji, J.-Y., Yang, F., Di Stefano, L., Herr, A., Moon, N.-S., Kwon, E.-J., Haigis, K.M., Näär, A.M., and Dyson, N.J. (2008). E2F1 represses beta-catenin transcription and is antagonized by both pRB and CDK8. Nature 455, 552-556.

# Eukaryotic cell lines

Policy information about cell lines

| | |
|---|---|
| Cell line source(s) | HEK293T (ATCC, cat# CRL-11268) |
| Authentication | No specific authentication of cell line was performed. |
| Mycoplasma contamination | Microscopy analysis did not reveal any suspicion of mycoplasma contamination. |
| Commonly misidentified lines (See ICLAC register) | Lines from ICALC register were not used in this study. |

# Animals and other organisms

Policy information about studies involving animals; ARRIVE guidelines recommended for reporting animal research

| | |
|---|---|
| Laboratory animals | Timed pregnant female CD-1 mice, Nestin-Cre mice, MADM-11GT/GT mice, MADM-11TG/TG mice, GFAP-GFP mice were used. Both female and male were used. Age used: E11.5, E12.5, E13.5, E14.5, E15.5, E16.5, E17.5 and P5. All animal procedures used in this study were performed in accordance with the protocol approved by the Institutional Animal Care and Use Committee of the Institute of Biophysics, Chinese Academy of Sciences. All mice had free access to food and water, were housed in the institutional animal care facility (SPF, at temperature of 21°C, relative humidity of 60%) with a 12hr light-dark schedule. |
| Wild animals | The study did not involve wild animals. |
| Field-collected samples | The study did not involve field-collected samples. |
| Ethics oversight | Mouse housing and experimental protocols in this study were in compliance with the guidelines of the Institutional Animal Care and Use Committee of the Institute of Biophysics, CAS. |

Note that full information on the approval of the study protocol must also be provided in the manuscript.

# Human research participants

Policy information about studies involving human research participants

| | |
|---|---|
| Population characteristics | We used human fetal hypothalamus. Gestational age was measured in weeks from the first day of the woman's last menstrual cycle to the sample collecting date. |
| Recruitment | Beijing Anzhen Hospital was in charge of recruiting donors for this research. The patients decided to have an abortion first, and then they were asked whether they would agree to donate the fetal tissues to this study. The de-identified human fetal tissue samples were collected after the donor patients signing informed consent document. The tissue collection and research protocols were approved by the Reproductive Study Ethics Committee of Beijing Anzhen Hospital and the institutional review board (ethics committee) of the Institute of Biophysics. |
| Ethics oversight | Research protocols were approved by the Reproductive Study Ethics Committee of Beijing Anzhen Hospital and the institutional review board (ethics committee) of the Institute of Biophysics. |

Note that full information on the approval of the study protocol must also be provided in the manuscript.
